# Predictive Rules of Efflux Inhibition and Avoidance in Pseudomonas aeruginosa

**DOI:** 10.1128/mBio.02785-20

**Published:** 2021-01-19

**Authors:** Jitender Mehla, Giuliano Malloci, Rachael Mansbach, Cesar A. López, Ruslan Tsivkovski, Keith Haynes, Inga V. Leus, Sally B. Grindstaff, Robert H. Cascella, Napoleon D’Cunha, Liam Herndon, Nicolas W. Hengartner, Enrico Margiotta, Alessio Atzori, Attilio V. Vargiu, Pedro D. Manrique, John K. Walker, Olga Lomovskaya, Paolo Ruggerone, S. Gnanakaran, Valentin V. Rybenkov, Helen I. Zgurskaya

**Affiliations:** a Department of Chemistry and Biochemistry, University of Oklahoma, Norman, Oklahoma, USA; b Department of Physics, University of Cagliari, Cagliari, Italy; c Theoretical Biology and Biophysics Group, Los Alamos National Laboratory, Los Alamos, New Mexico, USA; d Qpex Biopharma, San Diego, California, USA; e Department of Pharmacology and Physiology, St. Louis University, School of Medicine, St. Louis, Missouri, USA; f Department of Chemical Engineering, Massachusetts Institute of Technology, Cambridge, Massachusetts, USA; McMaster University

**Keywords:** *Pseudomonas aeruginosa*, antibiotic resistance, machine learning models, multidrug efflux, outer membrane

## Abstract

Antibiotic-resistant bacteria rapidly spread in clinical and natural environments and challenge our modern lifestyle. A major component of defense against antibiotics in Gram-negative bacteria is a drug permeation barrier created by active efflux across the outer membrane. We identified molecular determinants defining the propensity of small peptidomimetic molecules to avoid and inhibit efflux pumps in Pseudomonas aeruginosa, a human pathogen notorious for its antibiotic resistance. Combining experimental and computational protocols, we mapped the fate of the compounds from structure-activity relationships through their dynamic behavior in solution, permeation across both the inner and outer membranes, and interaction with MexB, the major efflux transporter of P. aeruginosa. We identified predictors of efflux avoidance and inhibition and demonstrated their power by using a library of traditional antibiotics and compound series and by generating new inhibitors of MexB. The identified predictors will enable the discovery and optimization of antibacterial agents suitable for treatment of P. aeruginosa infections.

## Abstract (precis)

Efflux pump avoidance and inhibition are desired properties for the optimization of antibacterial activities against Gram-negative bacteria. However, molecular and physicochemical interactions defining the interface between compounds and efflux pumps remain poorly understood. We identified properties that correlate with efflux avoidance and inhibition, are predictive of similar features in structurally diverse compounds, and allow researchers to distinguish between efflux substrates, inhibitors, and avoiders in P. aeruginosa.

## INTRODUCTION

Antibiotic resistance is a global threat expected to cause an estimated 300 million premature deaths by 2050 (1–3). Bacteria have evolved with both intrinsic and acquired resistance mechanisms to protect themselves from antimicrobial agents, leading to inefficacy of almost all available antibiotics and challenging the treatment options available against bacterial infections (4, 5). As a proxy for especially intractable infections, we focus in this study on Pseudomonas aeruginosa, a Gram-negative bacterium causing various hospital-acquired infections, including pneumonia (6), bloodstream infections (7, 8), and infections in cystic fibrosis patients (9). Multidrug-resistant P. aeruginosa clinical isolates are resistant to nearly all available antibiotics and have been identified as a serious threat by the Centers for Disease Control and Prevention (2). Antibiotic resistance is enabled by various molecular mechanisms that often act synergistically to protect bacteria against antibiotics (10–12). In P. aeruginosa, as in all Gram-negative bacteria, active efflux of antibacterial compounds by multidrug efflux pumps across the outer membrane (OM) permeability barrier is a major defense mechanism (13, 14).

Gram-negative cell envelopes are extremely effective in protecting cells from antibiotics due to the synergy between active efflux pumps and the OM, which creates a permeation barrier with enhanced efficacy (15–18). The system is characterized by a highly nonlinear behavior leading to efficient efflux of antibiotics that slowly permeate the OM even if they are poor substrates of efflux pumps in biochemical terms. As a result, the combined action of active efflux and the OM barrier protects cells from a broad range of compounds, including both “good” and “bad” substrates of efflux pumps.

The major challenge in developing new antibacterial agents is to improve their intracellular accumulation in Gram-negative bacteria, and consequently antibiotic efficacies, by modifying their structures (19–21). This improvement can be achieved either by increasing influx of antibiotics across the OM, by designing molecules that avoid the efflux mechanism, or by inhibiting active efflux pumps with efflux pump inhibitors (EPIs) (22–24). In addition, antibacterial and EPI properties can be combined within the same chemical structure to create potent drugs that inhibit their own efflux. Understanding how various physicochemical properties of compounds correlate with these different mechanisms of penetration is crucial for tackling effectively antibiotic resistance of P. aeruginosa and other critical and high-priority Gram-negative pathogens.

Recent studies demonstrated that heuristics or “rules” of accumulation differ for the penetration across the OM barrier and for the avoidance of efflux pumps, and at the lower level of rules hierarchy, bacterial species-specific differences in composition of the OM and efflux pump play an important role (13, 20, 21, 25). In Escherichia coli, higher levels of accumulation were proposed to be dictated mainly by the presence of primary amines and such physicochemical descriptors as amphiphilicity, low globularity (a quantity describing molecular shape with low values associated with planar-like molecules), and rigidity (26–29). These rules, however, are applicable mainly to E. coli and other enterobacteria, because they are dominated by permeability properties of general porins (25, 27). These porins are highly abundant in the enterobacterial OM and sift molecules based on their size, shape and electrostatic properties.

In contrast, the OM of P. aeruginosa carries an arsenal of substrate-specific porins that limit uptake to certain nutrients (30). In addition, P. aeruginosa constitutively expresses several efflux pumps with different substrate specificities. MexAB-OprM is the major constitutively expressed pump, which is largely responsible for intrinsic resistance to a variety of antibiotics under laboratory conditions (31–33). Substrate specificities of these transporters and the efflux constant, *K_E_*, which relates to the rates of active and passive efflux of a drug in the range of its low concentrations, are the major drivers of efflux avoidance rules (“efflux rules”) (15, 16). The unique features of P. aeruginosa and the lack of separation of the efflux and OM contributions in earlier models necessitate the quest for species-specific descriptors and rules of permeation.

In this study, we developed and validated new models that describe the avoidance and inhibition of active efflux in P. aeruginosa. To achieve this, we analyzed a series of 260 peptidomimetic compounds (Rempex compounds) active against P. aeruginosa. Rempex compounds possess two biological features of interest. First, they are EPIs that target MexAB-OprM and homologous efflux pumps and potentiate the antibacterial activity of levofloxacin and other antibiotics in P. aeruginosa cells (34–36). Second, they possess an intrinsic antibacterial activity and inhibit the growth of P. aeruginosa at certain concentrations. The compounds were optimized in medicinal chemistry programs specifically against P. aeruginosa and vary broadly in their properties (34, 37, 38). These features make Rempex compounds an excellent tool for deciphering predictive general rules of permeation and efflux avoidance in P. aeruginosa. We experimentally segregated contributions of active efflux from OM permeation and developed novel computational approaches to quantify molecular recognition by the inner membrane (IM) efflux transporter MexB and permeation through the OM. Finally, we applied machine learning algorithms to precisely identify descriptors of efflux substrates, inhibitors, and avoiders. The developed approach combines experimental data and predictors accounting for different physicochemical conditions allowing us screen for compounds with specific properties and to effectively guide drug design against P. aeruginosa infections.

## RESULTS AND DISCUSSION

### Rempex compounds readily permeate the outer membrane of P. aeruginosa and are substrates of efflux pumps.

**FIG 1:**
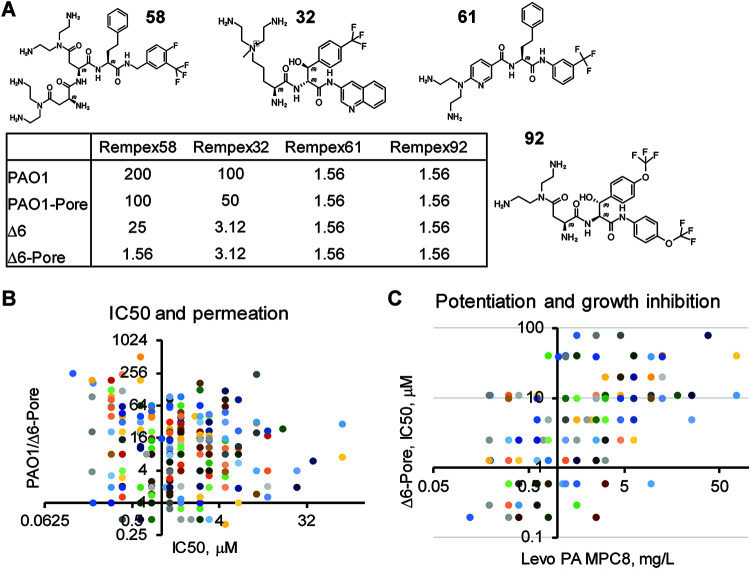
Antibacterial activities of Rempex compounds and their dependence on permeation and efflux. (A) Structures and activities (MICs, µg/ml) of representative Rempex compounds discussed in the text. The complete list of Rempex compounds and their activities are shown in Table S1. (B) Relationship between antibacterial activities of Rempex compounds (IC_50_) and the effect of the permeability barrier on these activities as expressed by the ratio PAO1/PΔ6-Pore (*r* = −0.16, *P* = 0.004). (C) Plot of levofloxacin potentiation of Rempex compounds (MPC_8_) as a function of their antibacterial activities (IC_50_) (*r* = 0.30, *P* = 0.01).

To follow the fate of compounds in cells and to identify descriptors associated with different permeation barriers, we first separated the contributions of the OM barrier and active efflux in measured activities of compounds. For this purpose, the bacterial growth-inhibitory activities for all Rempex compounds were analyzed in four P. aeruginosa strains: the wild-type strain, PAO1; the PΔ6 strain, lacking six efflux pumps (Δ*mexAB-oprM*, Δ*mexCD-oprJ*, Δ*mexXY*, Δ*mexJKL*, Δ*mexEF-oprN*, and Δ*triABC*); and their hyperporinated derivatives, PAO1-Pore and PΔ6-Pore, respectively (39). These strains were previously shown to differ dramatically in their susceptibilities to various classes of antibiotics because of differences in efflux proficiency and permeation across the OM (13, 15). To normalize to the differences in target inhibition potency among compounds, our key measured parameters were efflux ratios and OM barrier ratios, defined as the 50% inhibitory concentration for the parent/mutant (IC_50 parent_/IC_50 mutant_), for efflux mutants and hyperporinated mutants, respectively (see Table S1 in the supplemental material). More specifically, the IC_50_ ratios for PAO1/PAO1-Pore and PΔ6/PΔ6-Pore define the contribution of the OM barrier to the activities of compounds in the presence and absence of efflux, respectively. For the majority of compounds, these ratios were 1, suggesting that unlike most antibiotics (13, 28), Rempex compounds readily permeate the OM barrier, likely using the self-promoted uptake mechanism (40, 41). Activities of only a few compounds were slightly affected by the OM. Among them are compounds 58 and 46 (Fig. 1A), whose activity is enhanced by hyperporination of efflux-deficient PΔ6-Pore cells by 16- and 8-fold, respectively.

10.1128/mBio.02785-20.5 TABLE S1 Structures and activities of Rempex compounds. Download Table S1, XLSX file, 1.5 MB. Copyright © 2021 Mehla et al. 2021 Mehla et al. This content is distributed under the terms of the Creative Commons Attribution 4.0 International license.

The IC_50_ ratios of PAO1/PΔ6 and PAO1-Pore/PΔ6-Pore define the contribution of the efflux to the activities of compounds in the presence and absence of the OM barrier, respectively. Unlike with the OM ratios, the activities of ∼75% of compounds were significantly affected by active efflux (≥4-fold), with the highest ratios of 100 to 200 characteristic for such compounds as 71 and 32 (Fig. 1A). Thus, most Rempex compounds are substrates of P. aeruginosa efflux pumps.

The total contribution of the permeability barrier in activities of compounds is further elucidated by the IC_50_ ratio between PAO1 and PΔ6-Pore cells. Among compounds with the largest PAO1/Δ6-Pore ratios (128 to 256) are compounds 58 and 28, whose activity is affected by both active efflux and OM barrier (Fig. 1A).

Lastly, a comparison of the PAO1/PΔ6-Pore IC_50_ ratio and the IC_50_ in the PΔ6-Pore strain provides a measure of how the changes in the antibacterial activity on the target correlate with the permeation of a compound. There is a weak negative correlation (Pearson coefficient, *r=* −0.16, *P* = 0.004) between these two properties, suggesting that permeation is not the major limiting factor in the antibacterial activity of Rempex compounds (Fig. 1B).

### Rempex compounds inhibit efflux.

EPIs potentiate antibiotics’ activities by increasing their intracellular accumulation (37, 42). We analyzed the EPI activities using two different assays. We first analyzed the potentiation of the antibacterial activity of the antibiotic levofloxacin in PAM1032 cells overproducing the MexAB-OprM pump due to an *nfxB* mutation (37). Specifically, we identified the minimal potentiating concentration that reduces the MIC of levofloxacin by 8-fold (MPC_8_). The compounds varied broadly in their MPC_8_ values starting from 0.3 µM (compounds 17 and 32) and up to 190 µM (Fig. 1C). Overall, MPC_8_ values moderately correlated (*r =* 0.30, *P* = 0.01) with IC_50_ values in PΔ6-Pore cells. This result suggests that for some of the Rempex compounds, the potentiation of the levofloxacin activity is due to their antibacterial properties. However, certain compounds significantly deviate from this trend (Fig. 1C), pointing out that the EPI and antibacterial activities are independent from each other for these compounds.

In a second, bacterial growth-independent, assay, we analyzed the ability of compounds to inhibit efflux of the fluorescent probe Hoechst 33342 (HT), which diffuses slowly between leaflets of the cytoplasmic membrane and is pumped out from cells by efflux transporters. HT fluorescence increases 134- and 32-fold upon binding to DNA and lipids, respectively (15). We carried out the HT assay in hyperporinated PAO1-Pore cells to increase the permeation of both the probe and the compounds across the OM and to achieve efflux-saturating concentrations of compounds in the periplasm (15). We extracted the initial rates and intracellular steady-state concentrations of HT accumulation in cells from the time-dependent changes in HT fluorescence in the presence of increasing concentrations, *c*, of compounds (15).

**FIG 2:**
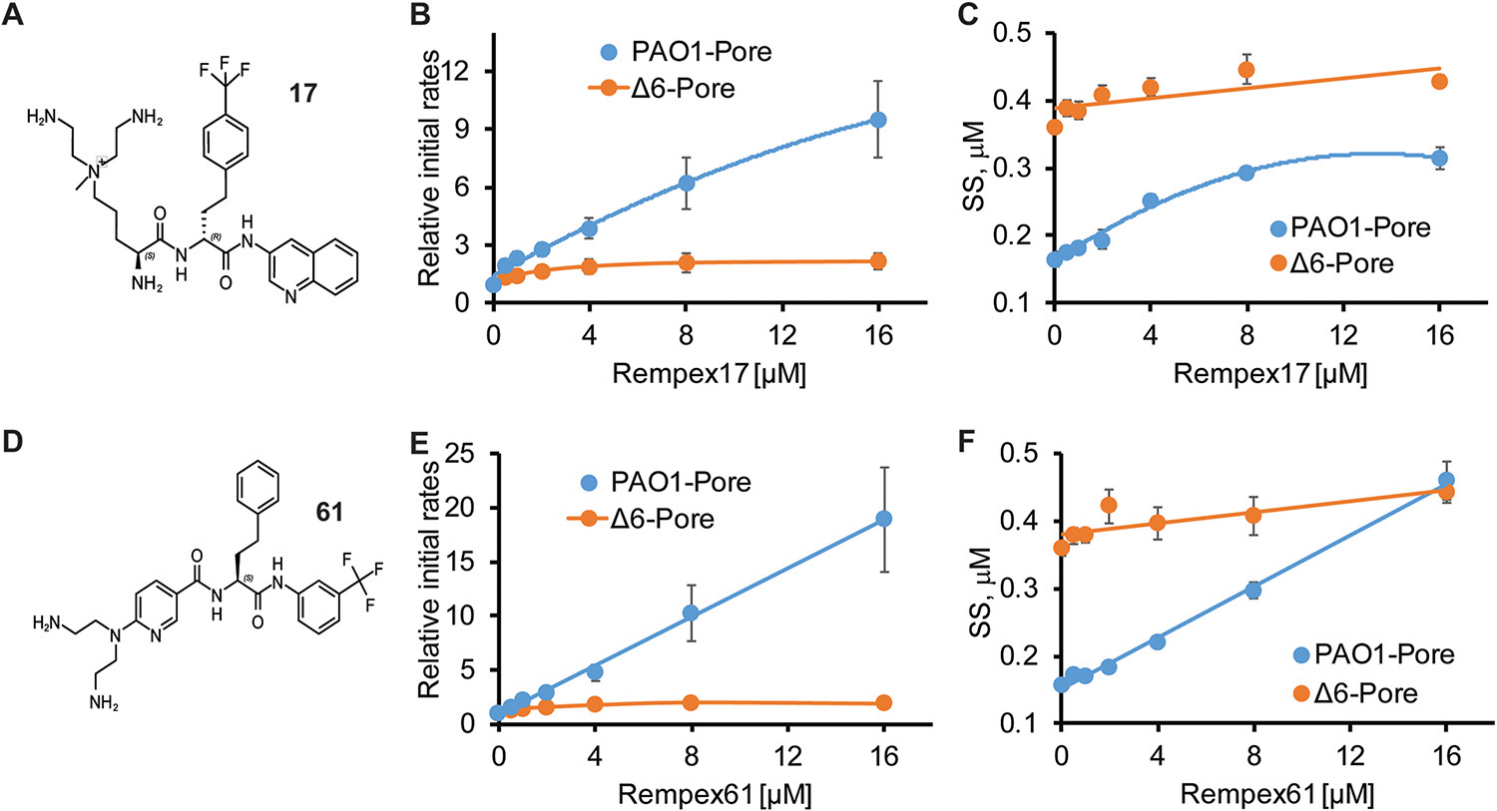
Efflux inhibition activities. (A and D) Structures of compounds 17 and 61. (B and C) Inhibition of efflux of the fluorescent probe bisbenzimide (Hoechst 33342 [HT]). Efflux-proficient PAO1-Pore cells and efflux-deficient PΔ6-Pore cells were incubated with 4 µM HT and increasing concentrations of 17, and changes in fluorescence of HT were recorded as a function of time. Fluorescence was converted into HT concentrations, and the kinetic curves were fitted into a double exponential equation to extract initial rates and the steady-state levels (SS) of the intracellular accumulation of HT. Initial rates were normalized to values at a zero concentration of compound 17, and both parameters are plotted as a function of the inhibitor concentration. (E and F) The same as panels B and C but for compound 61.

At *c* = 16 µM (the highest concentration of compounds used in the assay), about 50 compounds increased the intracellular concentration of HT by at least 2-fold. Some of the strongest inhibitors, (e.g., compounds 17 and 61) increased the initial rates of HT uptake by more than 10-fold (Fig. 2). This increase in the rates of HT accumulation was observed only in efflux-proficient PAO1-Pore cells, whereas few or no changes in rates were seen in efflux-deficient PΔ6-Pore cells. Furthermore, the increased rates of the probe accumulation in hyperporinated cells are specific to efflux inhibition, because in these cells the OM barrier does not limit the probe permeation. Thus, Rempex compounds inhibit active efflux specifically and independently from their ability to penetrate the OM of P. aeruginosa.

**FIG 3:**
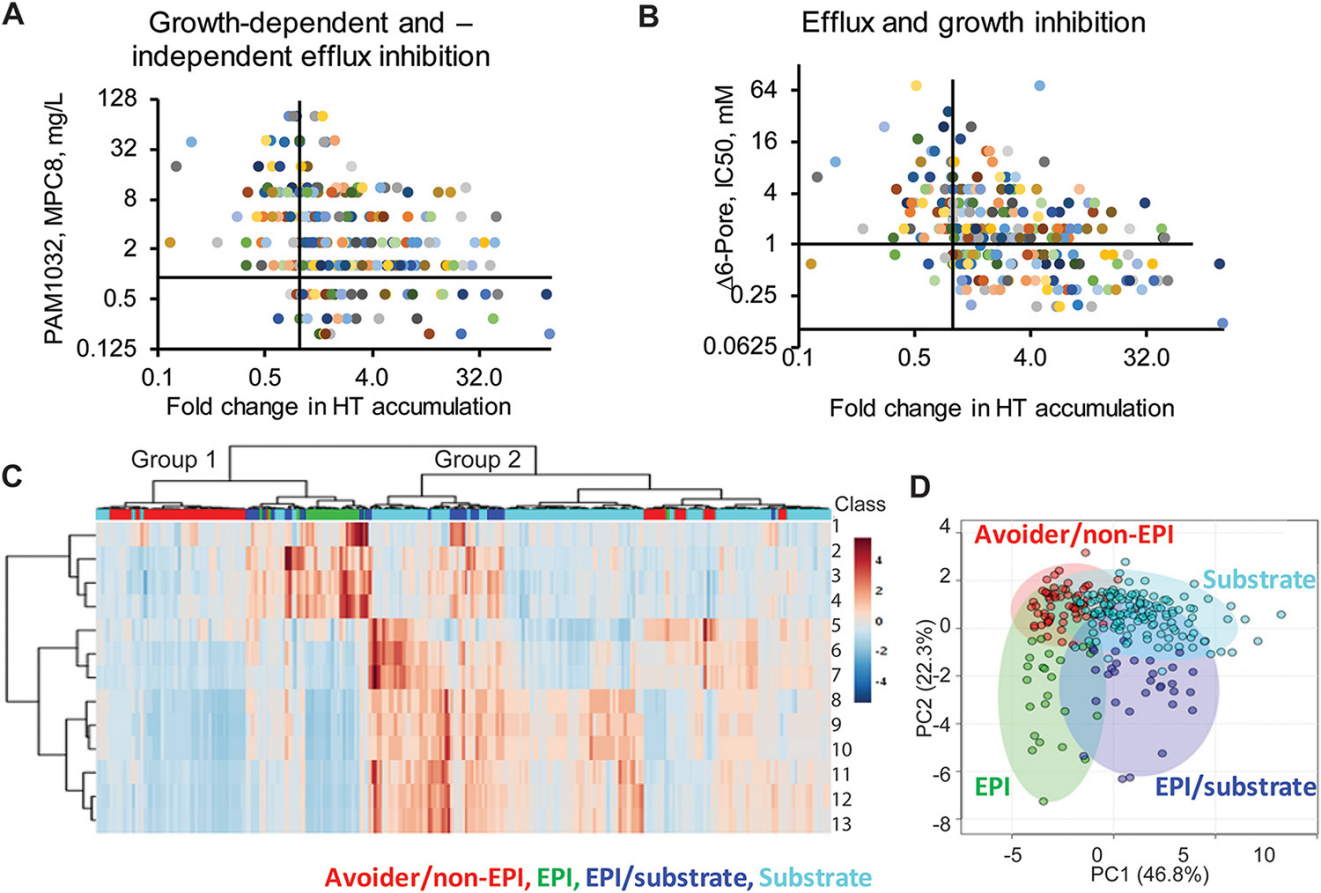
Relationships between activities of Rempex compounds. (A) Relationship between efflux inhibition activities expressed as MPC_8_ values (growth-dependent inhibition) and fold change in HT accumulation (growth-independent inhibition). (B) Relationship between EPI and antibacterial activities of compounds. (C) Hierarchical clustering of analyzed activities of compounds expressed as ratios (see also Materials and Methods). Class 1, ratio of initial rates of HT uptake at ratio of 0 to 16 µM (HT_16 μM_/HT_0 μM_); class 2, HT accumulation levels in the slow step (HT_16 μM_/HT_0 μM_); class 3, EPI_SS_, total HT accumulation ratio (HT_16 μM_/HT_0 μM_); class 4, fold difference in HT fluorescence (HT_16 μM_/HT_0 μM_); class 5, MIC_PΔ6_/MPC_8 PΔ6_; class 6, MIC_PAO1_/MPC_8 PA1032_; class 7, IC_50 PAO1_/MPC_8 PA1032_; class 8, MIC_PAO1_/MIC_PΔ6_; class 9, MIC_PAO1-Pore_/MIC_PΔ6-Pore_; class 10, MIC_PAO1_/MIC_PΔ6-Pore_; class 11, IC_50 PAO1_/IC_50 PΔ6_; class 12, IC_50 PΔ6-Pore_/IC_50 PAO1-Pore_; class 13, IC_50 PAO1_/IC_50 PΔ6-Pore_. The bar on the top is colored as follows: red, efflux avoiders and non-EPIs; green, EPIs; blue, compounds that are both EPI and substrates; cyan, substrates. (D) PCA plot with four classes of compounds colored as in panel C.

If both the bacterial growth-dependent and –independent assays measured the ability of compounds to inhibit active efflux, we would expect that the outcomes of the assays correlate with each other. Indeed, we found that the effect of Rempex compounds on the kinetics of HT accumulation correlates negatively, albeit weakly (*r =* −0.20, *P* = 0.0005), with their levofloxacin potentiating (MPC_8_) activities (Fig. 3A). Importantly, the most efficient inhibitors of HT efflux also have the lowest MPC_8_ values (Fig. 3B). Thus, both assays report on efflux inhibition, but additional factors also contribute.

As shown above, antibacterial and levofloxacin potentiating activities of compounds have a similar positive trend (*r* = 0.30, *P* = 0.01 [Fig. 1C]). In agreement, the activity in terms of the inhibition of HT efflux negatively correlates with the IC_50_s of compounds in PΔ6-Pore cells. However, this correlation is statistically weak (*r =* −0.11, *P* = 0.04), and many compounds deviate from it. Hence, the inhibition of HT efflux by Rempex compounds is defined by some properties that are independent from their antibacterial and potentiating activities.

### Efflux avoidance dominates clustering of compound activities.

Relationships between various activities of compounds were analyzed by hierarchical clustering. On the dendrogram (Fig. 3C), Rempex compounds separate into two large groups. The distinction between groups 1 and 2 mainly arises from the impact of efflux inactivation in bacterial growth inhibition assays. Group 1 primarily comprises compounds that are not affected by the inactivation of efflux (that is, they are not efflux substrates). Group 2 primarily comprises compounds that are strongly affected by the inactivation of efflux (that is, they are efflux substrates). Investigating further the subclusters of the two primary groups, we found that the compounds cluster according to their efficiencies in inhibition of HT efflux, as well as according to their antibacterial and levofloxacin potentiating activities.

Principal-component analysis (PCA) showed that between groups 1 and 2, four subgroups could be defined comprising compounds that are either effective inhibitors of HT efflux (green, EPI; blue, EPI/substrate) or do not have significant EPI activities (red, Avoider/non-EPI; cyan, Substrate). Although the subgroups lack a distinct boundary dividing them (Fig. 3D), the preponderance of points belonging to each of these subgroups falls into separate quadrants of the PCA plot. Thus, efflux inhibitory activities and efflux avoidance are not strongly interlinked, and these properties are associated with distinct compounds.

### Physicochemical, permeation, and MexB interaction descriptors of compounds.

To identify properties of compounds that correlate with their biological activities, we assembled several subsets of numerical descriptors and carried out an agglomerative clustering analysis to find the relationships between various descriptors belonging to either the same or different subsets.

**FIG 4:**
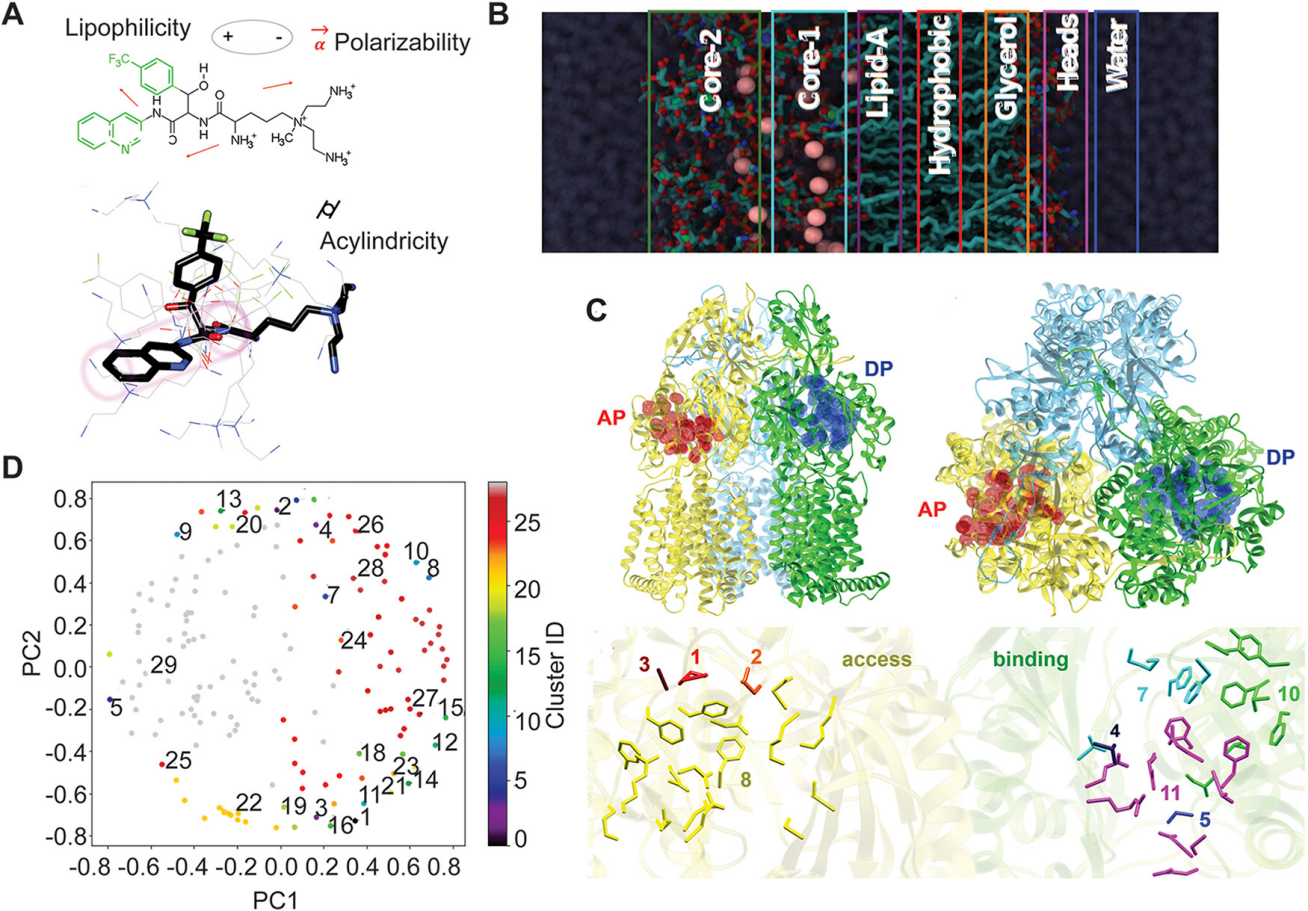
Physicochemical, permeation, and interaction descriptors. (A) Physicochemical descriptors illustrated on compound 32 as an example. (B) Representation of the OM model for calculating membrane permeability descriptors. For each drug, seven different MD simulations were performed, recapitulating the effect of the OM on drug translocation. Structurally, each slab corresponds to the following chemical regions along the OM (from top to bottom): Core-2, rhamnose and glucose; Core-1, heptose, keto-deoxyoctulosonate (KDO1 and KDO2), and *N*-acetylglucosamine (NAG1 and NAG2); Lipid-A, hydrophobic tails of the lipid A region; Hydrophobic, interface between the outer leaflet and the inner leaflet; Glycerol, glycerol moiety of the DPPE inner leaflet; Heads, ethanolamine and phosphates pertaining to DPPE head groups; Water, interfacial water region between the inner leaflet and the water bulk. (C) The top panel shows the front (left) and top (right) views of the MexB homotrimer. The three protomer conformations access, binding, and extrusion are colored in cyan, yellow, and green, respectively. The affinity sites deep binding pocket (DP) and access pocket (AP) are represented in blue and red, respectively. The bottom panel shows clusters of contacts with MexB amino acid residues in the AP and DP sites. The cluster number and its residues are shown in different colors. (D) PCA plot of clusters comprising all descriptors.

The chemical structures of compounds, their physical properties, and their interactions with the solvent were represented by 73 physicochemical descriptors (see Table S2 and Table S3, “Physico-chemical properties” column, in the supplemental material). These descriptors are frequently used in quantitative structure-activity relationship (QSAR) studies (QSAR descriptors), along with those derived from quantum-mechanical (QM) calculations (QM descriptors) and microsecond-long molecular dynamics (MD) simulations in explicit water solution (MD descriptors) (Fig. 4A). (43).

10.1128/mBio.02785-20.6 TABLE S2 Definitions of descriptors. Download Table S2, DOCX file, 0.03 MB. Copyright © 2021 Mehla et al. 2021 Mehla et al. This content is distributed under the terms of the Creative Commons Attribution 4.0 International license.

10.1128/mBio.02785-20.7 TABLE S3 Composition of clusters. Download Table S3, XLS file, 0.07 MB. Copyright © 2021 Mehla et al. 2021 Mehla et al. This content is distributed under the terms of the Creative Commons Attribution 4.0 International license.

Diffusion of compounds across membranes was represented by 35 permeation descriptors (Table S3, “Permeation descriptors” column). Rempex compounds readily permeate the OM of P. aeruginosa, as seen from the values of the OM ratios—close to 1 for most of them (Fig. 1A). To generate the permeation descriptors, compounds were placed into seven different layers of the OM model corresponding to the outer and inner cores of lipopolysaccharide (LPS; cores 2 and 1), lipid A headgroup, hydrophobic core of the bilayer, glycerol layer of the inner leaflet, the phospholipid layer headgroup, and water (Fig. 4B). For each simulation, the following molecular descriptors were evaluated: (i) membrane-ligand interaction energy, (ii) number of hydrogen bonds between a compound and its surrounding water shell, (iii) number of hydrogen bonds between the ligand and the surrounding OM environment, (iv) lateral mean squared displacement, (v) ligand hydration shell, and (vi) ligand cumulative entropy (Table S3, “Permeation descriptors” column).

Since ∼75% of Rempex compounds are efflux substrates, they likely interact with MexB, the IM transporter of the MexAB-OprM efflux pump. MexB is responsible for recognition, binding, and transport of substrates with very different molecular properties (31–33, 35, 42). As expected, we found that, with a few exceptions, most of the compounds bound the purified MexB in surface plasmon resonance (SPR) assays, with equilibrium dissociation constant (*K_D_*) values ranging from the sub- to the mid-micromolar range (see Fig. S1 in the supplemental material). To quantify interactions of Rempex compounds with MexB at a molecular level, we carried out ensemble docking calculations using available X-ray crystal structures of MexB (44, 45), along with a few conformations extracted from microsecond-long MD simulations (46). The MexB trimer is suggested to functionally rotate through three main conformations—access (A), binding (B), and extrusion (C)—which enable access, binding, and extrusion of substrates from cells, respectively (23). Rempex compounds were docked to the two major putative substrate binding pockets of MexB, as known from X-ray crystallography: (i) the access pocket (AP) of the access monomer and (ii) the deep binding pocket (DP) of the binding monomer (Fig. 4C) (47). Docking calculations yielded about 60 descriptors of binding of Rempex compounds inside the AP and DP sites of MexB (Table S2, docking descriptor definitions). These descriptors include average binding affinities and the total number of contacts between compounds and specific residues lining the two analyzed pockets (Fig. 4C; Table S3, “MexB docking descriptors” column).

10.1128/mBio.02785-20.1 FIG S1 Interactions with MexB and antibacterial activities of representative compounds. (Top row) Compounds 17, 46, and 89 were injected over immobilized MexB at indicated concentrations. The collected sensograms (colors) were fitted into 1:1 and two-state kinetic models (black lines) to extract on and off rates and the equilibrium dissociation constant, *K_D_*. For compound 17, *K_D_* was 24.2 µM, and for compound 89, it was found to be 10.9 µM. No significant binding to MexB was found for compound 46. (Middle row) Structures of indicated compounds. (Bottom row) MICs (mg/liter) in four strains of P. aeruginosa. Both compounds 17 and 89 are substrates of MexB, as seen from the reduced MICs in the PΔ6 strain. In contrast, compound 46 is not a substrate of MexB. Download FIG S1, TIF file, 0.1 MB. Copyright © 2021 Mehla et al. 2021 Mehla et al. This content is distributed under the terms of the Creative Commons Attribution 4.0 International license.

We next performed a cluster analysis on these three sets of descriptors separately and on all 174 descriptors combined (“all descriptors”) to identify possible correlations among different properties (Fig. 4D; Table S3, “All properties” column). We indeed identified 29 clusters, most of which had a clear association and included descriptors related to specific properties of compounds: molecular symmetry, size, charge and polarity, and number of rings (Table S3). A comparison with the results of clustering on property-specific subsets of descriptors (physicochemical, docking, and permeation) showed that for the most part, descriptors of physically related properties (e.g., size, number of rings, or entropy of permeation) are clustered together, regardless of the subset of descriptors considered. Thus, the identified clusters appear to be reliable and generally consistent with physical intuition.

### Derivation of predictive models of permeation and efflux in P. aeruginosa.

To assess the importance of different descriptors, we trained a linear predictive model based on experimental measurements and determined the relative importance of different predictors through the relative weight of their coefficients. To fit this model, we employed representative descriptors from the clusters discussed above. Seven distinct variables derived from experimental ratios were used for model outputs (see Table S4 and supplemental methods in Text S1 in the supplemental material): efflux = IC_50 PΔ6-Pore_/IC_50 PAO1-Pore_, permeation = IC_50 PΔ6-Pore_/IC_50 PΔ6_, EPI-1 =* g*(MIC_PAO1_/MPC_8 PA1032_), EPI-2 =* g*(MIC_PΔ6-Pore_/MPC_8 PA1032_), EPI_MPC_ = *g*(IC_50 PAO1_/MPC_8 PA1032_), and EPI_SS_ = SS_16 µM_/SS_0 µM_. SS_concn_ (e.g., SS_16 μM_) refers to the steady-state HT accumulation ratio at that concentration, and fold difference is the fold difference in HT fluorescence (HT_16 μM_/HT_0 μM_).

10.1128/mBio.02785-20.8 TEXT S1 Supplemental methods. Download Text S1, DOCX file, 0.02 MB. Copyright © 2021 Mehla et al. 2021 Mehla et al. This content is distributed under the terms of the Creative Commons Attribution 4.0 International license.

10.1128/mBio.02785-20.8 TABLE S4 Modeling experiments and parameters. Download Table S4, DOCX file, 0.02 MB. Copyright © 2021 Mehla et al. 2021 Mehla et al. This content is distributed under the terms of the Creative Commons Attribution 4.0 International license.

To identify the most generalizable descriptors, we performed feature selection employing regression analyses for (i) all descriptors and (ii) the following specific subsets: LigMexB descriptors (all except permeation descriptors), Lig descriptors (QSAR, QM, and MD descriptors), docking descriptors, and permeation descriptors. (See Fig. S2 in the supplemental material for an overview of the procedure.) The top descriptors were then used to fit a model able to predict whether a given compound based on its specific descriptors is a strong or weak (i) membrane permeator, (ii) efflux avoider, or (iii) efflux inhibitor.

10.1128/mBio.02785-20.2 FIG S2 Algorithm to identify top descriptors. Download FIG S2, TIF file, 0.2 MB. Copyright © 2021 Mehla et al. 2021 Mehla et al. This content is distributed under the terms of the Creative Commons Attribution 4.0 International license.

**FIG 5:**
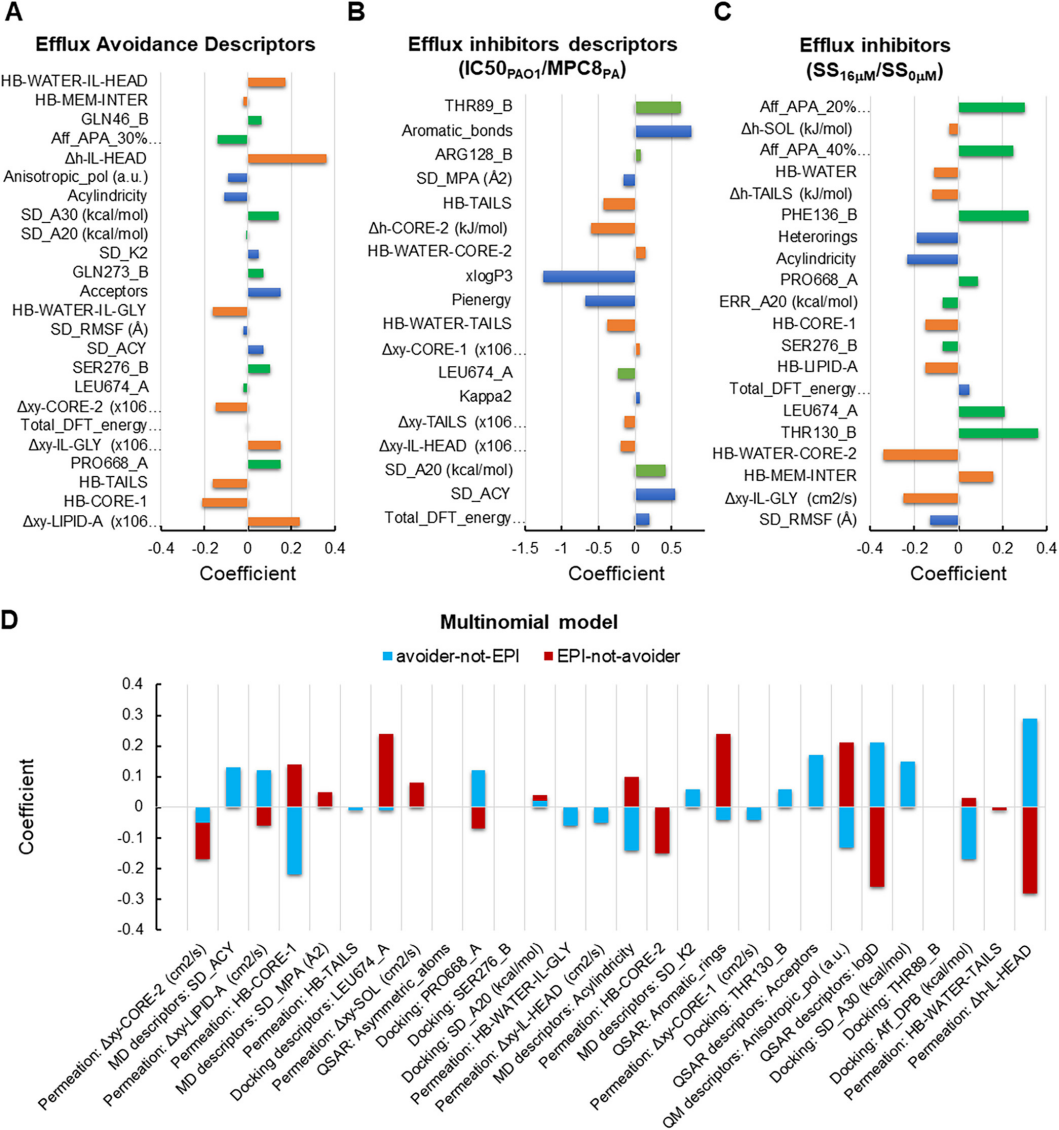
Top descriptors correlating with and distinguishing between efflux avoiders and inhibitors. (A) Efflux avoidance descriptors. Orange bars, permeation descriptors; green bars, docking descriptors; blue bars, physicochemical descriptors. (B) Efflux inhibitor descriptors (IC_50 PAO1_/MPC_8 PA1032_). Bars are colored as in panel A. (C) Efflux inhibitor descriptors (SS_16 µM_/SS_0 µM_). Bars are colored as in panel A. (D) Descriptors that distinguish between avoiders and inhibitors. Descriptor definitions are in Table S3.

Overall, we found that for the permeation, efflux, EPI_MPC_, and EPI_SS_ experimental ratios (four ratios out of seven analyzed), the LigMexB and “all descriptors” subsets of descriptors generated the best-performing models (see Fig. S3 in the supplemental material). We focused on a set of final models fitted with “all descriptors” (Fig. 5), because these descriptors incorporate molecular-level interactions and diffusion of compounds across both the OM and the IM. These final models were assessed and found to be well performing through the metrics of enrichment, precision, and recall as a function of both probability and ranking (see Text S1, supplemental methods, and Fig. S4 in the supplemental material).

10.1128/mBio.02785-20.3 FIG S3 Performance of models using different subsets of descriptors. We randomly resampled with replacement the unused 25% of the data 100 times and assessed model performance through a number of different metrics. (A) Accuracy is the fraction of correct predictions [(TN + TP)/(TN + TP + FN + FP)], where TN is the number of true negatives, TP is the number of true positives, FN is the number of false negatives, and FP is the number of false positives. (B) Balanced accuracy is the accuracy with each class weighted by the inverse of its frequency. (C) Recall and (D) precision of positives (class 1), where precision = TP/(TP+FP) and recall = TP/(TP+FN). (E) The F1 score, which is the weighted average of precision and recall, F1 = 2 × (precision × recall)/(precision + recall), for both classes. Balanced accuracy is somewhat more useful for our data sets, as in almost all cases we have a greater number of negative examples than positive ones. Precision measures how many items that were found to be hits were true hits—a higher precision means less wasted experimental effort on items suggested to be hits that are not truly hits. Recall measures how many of the overall hits were actually found by the classifier—a higher recall means less chance of missing good drugs when moving to experimental assessment. Abbreviations of *x*-axes: All, model with all 174 descriptors; md, model based on the LigMexB subset comprising the QSAR, QM, MD, and docking descriptors; perm, model with permeation descriptors only; pchem, model with the Lig subset of QSAR, QM, and MD descriptors; dock, model with docking descriptors only. Legend: efflux, IC_50 PAO1-Pore_/IC_50 PΔ6-Pore_; permeation, IC_50 PΔ6_/IC_50 PΔ6-Pore_; EPI-1, MIC_PAO1_/MPC_8 PA1032_; EPI-2, MIC_PΔ6-Pore_/MPC_8 PA1032_; EPI-3, IC_50 PAO1_/MPC_8 PA1032_; steady state, SS_16 µM_/SS_0 µM_; fold-difference, HT_16 µM_/HT_0 µM_. Download FIG S3, TIF file, 2.0 MB. Copyright © 2021 Mehla et al. 2021 Mehla et al. This content is distributed under the terms of the Creative Commons Attribution 4.0 International license.

10.1128/mBio.02785-20.4 FIG S4 (Top left) Assessment of a binomial model of efflux avoidance properties. (A and C) Enrichment curves for the hit class versus (A) ranking and (C) probability. (B and D) Precision and recall curves for the hit class versus (B) ranking and (D) probability. In all cases, error bars represent the standard error of 100 bootstrap resampled runs: that is standard deviation/10. The enrichment at a particular ranking of *r* is the fraction of hits found of the top *r* % of predicted items minus the overall fraction of hits that could be found and divided by the total number of items. An enrichment of 2 at a ranking of 10%, for example, indicates that by considering the top 10% of predicted hits, we find 200% more than we would find by brute force search, corresponding to a halving of the search space. (Bottom left) Assessment of a binomial model of EPI_MPC_. (A and C) Enrichment curves for the hit class versus (A) ranking and (C) probability. (B and D) Precision and recall curves for the hit class versus (B) ranking and (D) probability. (Top right) Assessment of a binomial model of EPI_SS_. (A and C) Enrichment curves for the hit class versus (A) ranking and (C) probability. (B and D) Precision and recall curves for the hit class versus (B) ranking and (D) probability. Download FIG S4, TIF file, 1.6 MB. Copyright © 2021 Mehla et al. 2021 Mehla et al. This content is distributed under the terms of the Creative Commons Attribution 4.0 International license.

### Efflux avoidance and inhibition correlate with distinct molecular descriptors.

We next identified among the top descriptors those that correlate with experimental measurements by inspecting the corresponding coefficients in the binomial regression models. A positive (negative) coefficient implies that the descriptor positively (negatively) correlates with the outcome. The larger the magnitude of the coefficient, the more important it is to the outcome (Fig. 5).

### (i) Efflux avoiders.

We assumed molecules with efflux ratios IC_50 PΔ6-Pore_/IC_50 PAO1-Pore_ of ≤0.25 to be weak efflux avoiders, while we considered compounds with ratios IC_50 PΔ6-Pore_/IC_50 PAO1-Pore_ of ≥0.25 to be strong efflux avoiders (Table S4). The best model predicts positive correlations with the docking descriptors’ contacts with P668 of the AP and S276, Q273, and Q46 of the DP and the permeation descriptors of diffusion within lipid A and within the glycerol moieties of phospholipids (Fig. 5A). Also, an increase in the number of hydrogen bond acceptors (physicochemical descriptor) in a compound is correlated with strong efflux avoidance. On the other hand, increase in hydrogen bonding with water and lipid moieties of the membranes (permeation), more contacts with L674 in the AP site (docking), and increased acylindricity and anisotropic polarizability of compounds (physicochemical) appear to make them better efflux substrates (negative correlation).

### (ii) Efflux inhibitors.

From the two types of EPI assays, the growth-dependent IC_50 PAO1_/MPC_8 PA1032_ (EPI_MPC_) ratio and the growth-independent SS_16 µM_/SS_0 µM_ (EPI_SS_) ratio generate the best-performing models for efflux inhibitors (Fig. S4). For both of these ratios, the “good”/“bad” cutoffs were set at 0.5.

As in the case of efflux substrates, the top descriptors that discriminate between “good” and “bad” EPI_MPC_ are dominated by permeation descriptors. The best EPI_MPC_ ratios track descriptors indicative of slow diffusion within the phospholipid headgroups and lipid tails of the membrane (Δ*xy*) and lower hydrogen bonding with lipid moieties and with penetrating water (HB) (Fig. 5B). The dominance of these descriptors suggests that they represent unique features of compounds, perhaps related to the fact that MexB and similar pumps appear to capture their substrates from the lipid bilayers and a water-lipid interface (48, 49). The difference in hydrogen-bonding profiles between inhibitors and avoiders likely reflects the compound affinities to various layers of the OM model and their optimal positions in these layers. With respect to docking descriptors, more potent inhibitors have fewer contacts with L674 in the AP and more with T89 and R128 in the DP (Fig. 5B). Among physicochemical descriptors, the number of aromatic bonds, total density functional theory (DFT) energy and relative shape anisotropy Kappa2 increase with the potency of EPI_MPC_, whereas pi energy and lipophilicity (as expressed by xLogP3) decrease with increasing potencies (negative correlation) (Fig. 5B).

Interestingly, the top descriptors for the growth-independent EPI_SS_ are dominated by interactions with MexB (Fig. 5C). Average binding affinity to the AP as well as contacts with L674 and P668 in the same site correlate positively with the activity of EPI_SS_. In addition, more contacts with T130 and F136 in the DP correlate with higher EPI_SS_ activities. In contrast, seven out of eight top permeation descriptors negatively correlate with these ratios. Most of these descriptors are hydrogen bonding with polar moieties and water. Thus, decreasing hydrogen bonding propensity is expected to increase the activity of EPI_SS_ (Fig. 5C). Among the physicochemical features, the acylindricity and the number of heteroaromatic rings negatively correlate with EPI_SS_.

### (iii) Properties distinguishing between efflux avoiders and efflux inhibitors.

Finally, a combination of experimental ratios defining efflux avoidance (IC_50 PΔ6-Pore_/IC_50 PAO1-Pore_) and efflux inhibition (EPI_MPC_) and the same thresholds as used for the models above were employed to identify descriptors that may be useful in distinguishing these two properties, rather than being solely predictive of one or the other. Initially, we considered four possible classes: good avoidance and good inhibition (efflux ≥ 0.25, EPI_MPC_ ≥ 0.5) (GG), good avoidance but bad inhibition (efflux ≥ 0.25, EPI_MPC_ ≤ 0.5) (GB), bad avoidance but good inhibition (efflux ≤ 0.25, EPI_MPC_ ≥ 0.5) (BG), and bad avoidance and bad inhibition (efflux ≤ 0.25, EPI_MPC_ ≤ 0.5) (BB). Assessment of class balance shows that approximately 57% of compounds are in the BB class, 23% are in the GB class, 20% are in the BG class, and 0% are in the GG class. Due to the lack of examples in the fourth class, we trained a 3-class multinomial regression classifier. In this model, precision and recall for all three classes are good, as well as the enrichment for classes 1 and 2, which are of greater interest (approximately 2 to 3) (Fig. S4).

An inspection of the model parameters shows that there is little predictivity for the BB class (relying solely on its intercept and presumably not being class 1 or class 2). Hence, we analyzed which descriptors are related to GB (avoider-not-EPI) alone, which are related to BG alone (EPI-not-avoider), and which are related to both (Fig. 5D). Three permeation descriptors have the largest coefficients and clearly separate compounds belonging to these two classes: (i) diffusion within lipid A layer; (ii) hydrogen bonding with core 1 layer, and (iii) total energy of interactions with phospholipid headgroups. Compounds that are neither good substrates nor EPIs (avoider-not-EPI, GB) positively correlate with diffusion in lipid A and binding to phospholipid headgroups, but negatively with hydrogen bonding with core 1. In contrast, compounds that are both efflux substrates and inhibitors (EPI-not-avoider, BG) have inverse properties and show a positive correlation with hydrogen bonding in core 1 and a negative coefficient for diffusion in lipid A and binding to headgroups (Fig. 5D).

Three docking descriptors distinguish the two classes: contacts with (i) L674 and (ii) P668 in the AP and (iii) average affinity to the DP of MexB (Fig. 5D). Compounds avoiding efflux have fewer contacts with L674 and lower preference for the DP, but more contacts with P668. In contrast, substrates/inhibitors interact more with L674 of the AP and the DP and negatively trend with P668.

Finally, three physicochemical properties distinguish the two classes: (i) molecular shape as described by acylindricity, (ii) anisotropic polarizability (Anisotropic_pol), and (iii) partition coefficient (LogD) (Fig. 5D). The propensity to be a substrate/inhibitor increases with increasing acylindricity and anisotropic polarizability but decreases with increasing partition coefficient and lipophilicity.

Taken together, these results demonstrate that for the bacterial growth-dependent measurements, there is an interrelatedness between the propensities of a compound to be an EPI and to be recognized as an efflux substrate, suggesting that both rely on a similar set of compound properties. However, efflux pump avoiders and EPIs/substrates can be separated based on their molecular interactions with membranes and MexB, their shape, lipophilicity, and electrodynamic response properties.

### Models identify efflux avoiders and EPIs/substrates among structurally unrelated compounds.

We next tested whether our models can rank unrelated compounds based on their ability to avoid or inhibit efflux. For this purpose, we calculated the LigMexB subset of descriptors for a library of 674 molecules. The library comprised compounds of the Chembridge Diversity set with unknown antibacterial properties, known efflux inhibitors, and traditional antibiotics that were not part of the training or testing set for any of the regression models described above. We next used the developed efflux and EPI_ss_ models to identify which of these molecules might be expected to avoid or inhibit efflux.

**FIG 6:**
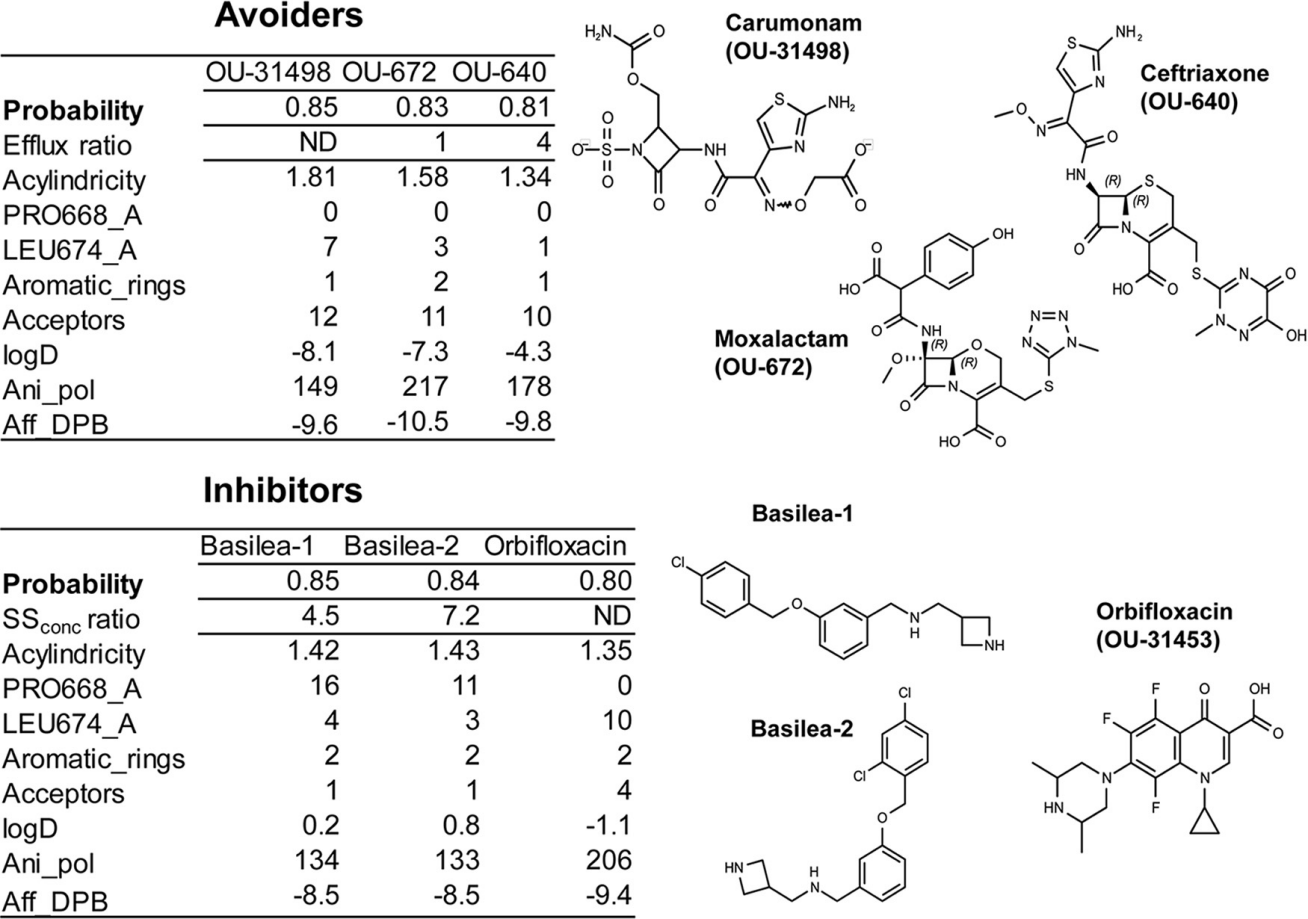
Structures, properties, and activities of the top predicted efflux avoiders and inhibitors. The tables show (i) the predicted probabilities for the compounds to avoid and to inhibit efflux, (ii) the measured efflux (IC_50 PΔ6-Pore_/IC_50 PAO1-Pore_) and SS_concn_ (SS_16 µM_/SS_0 µM_) ratios, and (iii) the selected calculated descriptors predicted to tune between efflux avoiders and inhibitors. (See Table S3 for a complete list of descriptors.)

The 15 top-ranked efflux avoiders (probability of ≥0.76) are dominated by antibiotics, including three monobactams, seven cephalosporins, and sulbenicillin from the penicillin class (Fig. 6; see Table S5 in the supplemental material). The remaining four species are from a previously reported series of compounds with EPI activities in *E.coli* (50). MIC measurements for 11 of the top compounds showed that 9 have MICs in at least one of the four P. aeruginosa strains. For most of these compounds, the efflux ratios MIC_PΔ6_/MIC_PAO1_ and MIC_PΔ6-Pore_/MIC_PAO1-Pore_ ranged between 1 and 0.25, showing that indeed efflux plays a negligible role in their antibacterial activities (Fig. 6).

10.1128/mBio.02785-20.9 TABLE S5 Top-ranked predicted efflux avoiders and EPIs. Download Table S5, DOCX file, 0.02 MB. Copyright © 2021 Mehla et al. 2021 Mehla et al. This content is distributed under the terms of the Creative Commons Attribution 4.0 International license.

Eleven compounds ranked high (probability of ≥0.75) to have EPI-like properties and were different from the predicted efflux avoiders (Fig. 6; Table S5). Among these top-ranked putative EPIs, three are antibiotics of the fluoroquinolone class and three are EPIs showing good potentiation for a range of antibiotics against both *E coli* and P. aeruginosa (52). We tested EPI compounds on the ability to inhibit efflux of HT, the activity used to generate the EPI_ss_ model, and we found that all three compounds increase the intracellular accumulation of HT by at least 4-fold (Fig. 6). Fluoroquinolones are intrinsically fluorescent and could not be tested in this assay. However, these antibiotics potentiate the activities of penicillins and carbapenems (53, 54), and efflux inhibition could play a role in this synergism.

Thus, predictive rules for efflux avoidance and inhibition identified using a series of Rempex analogs appear to be applicable to a broader chemical diversity of compounds.

### Models guide optimization of compounds for efflux inhibition.

To further validate the identified efflux inhibition “rules,” we applied them to a series of compounds that do not have EPI activities against P. aeruginosa. The previously reported OU-266 series acts on AcrA, the periplasmic component of the E. coli AcrAB-TolC efflux pump and potentiates activities of novobiocin in this bacterium but not in P. aeruginosa (50). Furthermore, unlike Rempex compounds (Fig. 1) and the top predicted efflux inhibitors from the test library (Fig. 6), this series does not have considerable antibacterial properties. We next generated a limited series of OU-266 derivatives (Test S1, supplemental methods) and used the identified predictive descriptors of efflux inhibition to optimize their EPI-like properties.

**FIG 7:**
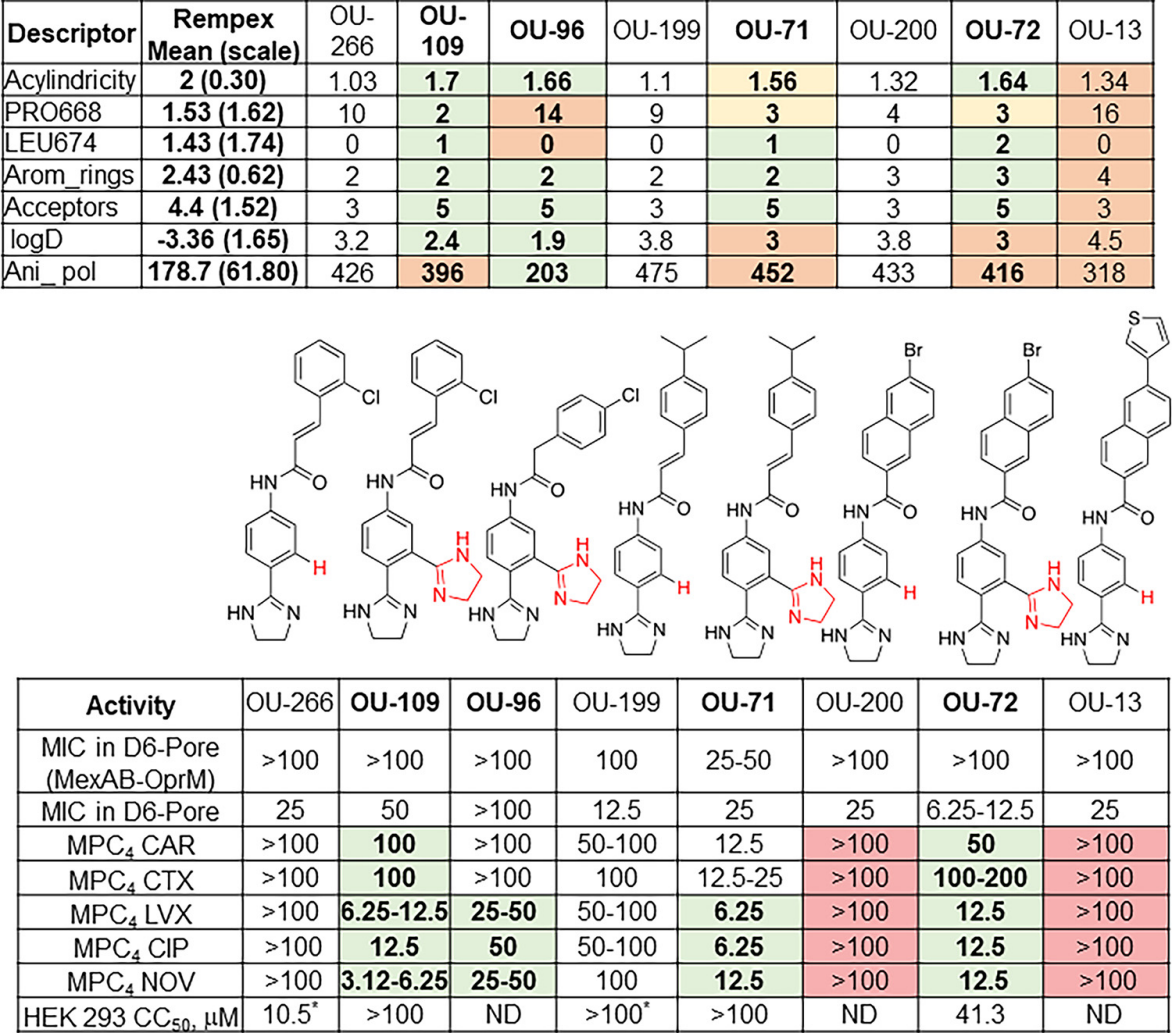
Structures, properties, and activities of EPIs optimized against MexAB-OprM. The top table shows the values of efflux inhibition predictors calculated for the indicated compounds. Values highlighted in green show desired changes, and those in yellow to orange show undesired changes. The bottom table shows the antibacterial (MIC) and EPI (MPC_4_ and SS_50 µM_/SS_0 µM_) activities of indicated compounds (CAR, carbenicillin; CTX, cefotaxime; LVX, levofloxacin; CIP, ciprofloxacin; NOV, novobiocin). Values highlighted in green show desired changes, and those in red show undesired changes. MICs were measured in efflux-deficient PΔ6-Pore cells and their complemented derivative carrying the plasmid-borne MexAB-OprM. MPC_4_ activities were analyzed in the complemented PΔ6-Pore(MexAB-OprM). CC_50_ (50% cytotoxic concentration) values with asterisks are from reference 50.

The major predictors of EPIs are their acylindricity (mean values for Rempex series, 2.00), anisotropic polarizability (mean, 178.7 Atomic Units [AU]), and the number of aromatic rings (mean, 2.43), all positively correlating with EPI activity, and the partition coefficient LogD (mean, −3.36), which correlates negatively (Fig. 7). In addition, interactions with L674 (mean, 1.43) and P668 (mean, 1.53) in the AP of MexB correlate with EPI-like properties positively and negatively, respectively (Fig. 5D). The properties of OU-266 notably deviate from the mean values calculated for Rempex compounds, but the molecule is asymmetric, with a hydrophobic and a polar terminus reminiscent of some of the features seen in Rempex compounds (Fig. 7). The addition of the second dihydroimidazoline ring in OU-109 aligned several of the top properties with the desired values. In particular, the acylindricity, the number of H-bond acceptors, and the contacts with P668 in MexB all moved into the optimal range. These changes led to a potent EPI activity against MexAB-OprM, as seen from the MPC values of OU-109 in combinations with the fluoroquinolones levofloxacin and ciprofloxacin, novobiocin, and different β-lactams (Fig. 7). Replacing 2-chlorophenyl with 4-chlorophenyl in OU-96 reduced anisotropic polarizability to the desired level and further reduced LogD, but these changes increased undesired contacts with P668 and, as a result, reduced EPI activity. The isopropylbenzene group increased hydrophobicity of OU-71 and OU-199 and enhanced their antibacterial activity without significant improvement of their EPI potencies. On the hydrophobic terminus of OU-266, the chlorophenyl can be substituted with a bromonaphthalene moiety in OU-72 without significant loss of EPI activity. However, this substitution enhanced the antibacterial activity and efflux of the compound by MexAB-OprM, as seen from the MICs of 6.25 to 12.5 µM in PΔ6-Pore cells and the lack of growth inhibition in PΔ6-Pore(MexAB-OprM). Further increasing the aromaticity in OU-1 shifted their LogDs and contacts with both P668 and L647 into undesired areas, leading to the loss of EPI properties (Fig. 7).

Thus, the top predictors of efflux inhibition discovered in this study can effectively guide the further development of compounds for efficient efflux inhibition in the challenging pathogen P. aeruginosa.

### Conclusions.

• Intracellular accumulation predictors generated using the model E. coli species have limited utility in optimization of antibacterial activities against P. aeruginosa, because of the powerful active efflux and the permeability barrier of the OM of this species.
• Interactions of compounds with the major efflux pump MexB and with the different layers of the OM of P. aeruginosa can be converted into numerical descriptors. In combination with traditional physicochemical properties of compounds, these descriptors can be used in modeling of efflux avoidance and permeation in P. aeruginosa.
• Antibacterial and efflux inhibitory activities of compounds correlate weakly and can be separated using bacterial growth-independent efflux inhibition assays. The two activities correlate with different sets of descriptors.
• Efflux ratios are reliable reporters of the propensity of compounds to avoid or to be captured by efflux pumps. Interactions with membranes, specific residues of MexB in AP, and the affinity to DP dominate efflux avoidance predictors likely reflect the contributions of specific residues to affinities of compounds to the substrate binding sites of MexB.
• Growth-dependent and -independent efflux inhibitory activities correlate with each other, albeit weakly, suggesting that they report on different properties of compounds. These properties correlate with different descriptors.
• Permeation predictors are prominent in both efflux inhibition and avoidance models, suggesting that these predictors represent properties of compounds that are not rendered by MexB docking and physicochemical descriptors. Possibly, these descriptors reflect the ability of MexB and similar pumps to capture their substrates from the lipid bilayer and at the water-lipid bilayer interface.
• The majority of Rempex compounds efficiently permeate the OM of P. aeruginosa*—*presumably by the self-promoted uptake mechanism—and their activities are only weakly affected by the OM barrier. Alternative libraries of compounds are needed to generate reliable models for OM permeation.
• Efflux avoidance and inhibition models are predictive of such properties among unrelated compounds, and the two models select different chemical classes of compounds. These models can be useful for *in silico* prefiltering of large compound libraries for the desired properties.
• Model-based optimization of efflux inhibitory activities leads to gain in antibiotic potentiation activities against P. aeruginosa.

## MATERIALS AND METHODS

### Chemicals and strains used.

All strains used were described previously (13, 28). The cells were grown in Luria-Bertani (LB) broth (10 g/liter tryptone, 5 g/liter yeast extract, and 5 g/liter NaCl) at 37°C with shaking. Rempex compounds were generated in the discovery/optimization campaign by Rempex Pharmaceuticals and provided by Qpex Biopharma. The EPI compounds in Fig. 6 and Table S5 were discovered, developed at, and provided by Basilea Pharmaceutica International, Ltd.

Antibacterial activities were tested using a 2-fold serial dilution broth assay as described previously (13). The antibacterial activities are expressed as MIC (defined as at least 90% of growth inhibition) and IC_50_.

The kinetics of Hoechst accumulation was analyzed as described previously in a temperature-controlled microplate reader (Tecan Spark 10M) in a fluorescence mode (15). Compounds were prescreened for possible interference with Hoechst fluorescence, and those compatible were further analyzed to establish concentration dependencies. The kinetic analysis was performed using a MatLab program as described previously (15).

### MexB purification and surface plasmon resonance assays.

MexB was purified from P. aeruginosa PAO1 cells harboring pMexB plasmid (55) using Cu^2+^ metal affinity chromatography as described previously (39, 56). Surface plasmon resonance (SPR) experiments were performed using a Biacore T200 (GE Healthcare) equipped with a research-grade CM5 S2 sensor chip. The purified MexB was immobilized by amino coupling. The immobilization and subsequent binding experiments were conducted in a running buffer containing 25 mM HEPES-NaOH (pH 7.0), 150 mM NaCl, and 0.2% Triton X-100 as described previously (56). Rempex compounds were screened for binding to the immobilized MexB at a concentration of 25 µM, followed by kinetic analysis of a selected subset of compounds at six different concentrations. Each compound/analyte was injected over the ligand and reference flow cells simultaneously at a flow rate of 30 μl/min and at a temperature of 25°C. The complex was allowed to associate and dissociate for 20 to 30 s and 150 s, respectively. The data were fit into a simple 1:1 binding (interaction) model or two-state kinetic model using the global data analysis for the association and dissociation rate constants *k_a_* and *k_d_*, respectively, and *R*_max_ available within Biacore Insight Evaluation software.

### QSAR, QM, and MD calculations.

For each compound, we generated the 2D structure data file (SDF format) and the protonation/charge state most populated at physiological pH 7.4 using the MOE package (57). We then used the ChemAxon’s Marvin suite of programs (58) to obtain 1-2-3D descriptors commonly used in QSAR studies, such as number of heavy atoms, isoelectric point, van der Waals volume and surface, number of rotatable bonds, number of H-bond donors/acceptors, etc. (Table S2). These descriptors include LogP values obtained with the XLOGP3 program (59). The configuration of the major microspecies has been used as an input to QM calculations performed with the Gaussian16 package as described in previous work (43). We optimized the ground-state structure employing a polarizable continuum model (60) as to mimic the effect of water solvent particularly to avoid formation of strong intramolecular H-bonds. To confirm the geometry obtained to be a global minimum on the potential energy surface, we performed full vibrational analyses, obtaining real frequencies in all cases. On the optimized geometry, we then performed single-point energy calculations in vacuum to generate the atomic partial charges fitting the molecular electrostatic potential. Under the constraint of reproducing the electric dipole moment of the molecule, we used the Merz-Kollman scheme (61) to construct a grid of points around the molecule. Atomic partial charges were then generated through the two-step restrained electrostatic potential method (62) implemented in the AnteChamber package (63). Using this program, we derived general Amber force field (GAFF) parameters (64). QM descriptors associated with the ground-state optimized structure include static polarizabilities, frontier molecular orbital energies, permanent dipole moment, and rotational constants. For each compound, we performed 1-μs-long all-atom MD simulation in explicit water solution (0.1 M KCl) using the Amber18 package as described before (43). From MD simulations, we obtained structural and dynamic features of the compounds investigated by means of the PTRAJ and CPPTRAJ programs of Amber18 (65). The number and population of structural clusters were determined using a hierarchical agglomerative algorithm (66).

### Ensemble docking to MexB.

All molecular docking calculations were performed using the software AutoDock Vina (67), implementing a stochastic global optimization approach. The program was used with default settings except for the exhaustiveness (giving a measure of the exhaustiveness of the local search), which was set to 1,024 (default of 8). Protein and ligand input files were prepared with AutoDock Tools (68). Flexibility of both docking partners was considered indirectly by using the ensemble of conformations. In particular, for each compound we used 10 different cluster representatives extracted from MD simulations in explicit water solution, while for MexB, we considered 6 conformations, including available X-ray crystal structures (PDB ID no. 2V50, 3W9I, and 3W9J) (44, 45) and MD snapshots extracted from MD simulations (46). For each docking run, we retained the top 10 docking poses. We performed two sets of guided docking runs into the two major substrate binding pockets of MexB: the access pocket of the access monomer (AP) and the deep binding pocket of the binding monomer (DP). In each case, the docking search was performed within a cubic volume of 40 by 40 by 40 Å^3^ centered in the center of mass of the pocket. The interaction between each compound and MexB was quantified by means of a statistical analysis of all putative binding poses, yielding about 60 descriptors. These descriptors include average binding affinities (predicted according to the docking scoring function) as well as the total number of contacts with single residues lining the two pockets (see Table S2).

### Permeation descriptors of interactions with the OM.

Initial coordinates of the P. aeruginosa OM were downloaded (51). The model has been parameterized in line with the GLYCAM force field (69), and parameters are adapted to run in the GROMACS (70) molecular dynamics engine. The OM model consists of an inner leaflet composed of 1,2-dipalmitoyl-*sn*-glycero-3-phosphoethanolamine (DPPE) and an outer leaflet composed of a truncated LPS structure. The membrane is fully solvated using the TIP3P water model (71), and anionic charges in the LPS molecules are counterbalanced with Ca^2+^ cations. A schematic representation of the model is provided in Fig. 5, and its parameterization was described previously (72). Similarly, parameters for drug molecules, derived as described above (43), were consistently adapted from the general Amber force field (GAFF) (64) and transformed into GROMACS input files using the AnteChamber PYthon Parser interfacE (ACPYPE) tool (73).

To extract the molecular descriptors of drug permeation across the OM membrane, each drug was placed into seven different molecular environments corresponding to specific regions along the direction perpendicular to the OM (Fig. 4). These regions were explicitly selected in order to cover the influence of both the inner (DPPC) and outer leaflet (LPS) of the OM. Thus, seven independent simulations per drug were necessary in order to recapitulate the influence of the OM into the permeation process. The whole procedure was automated via a series of bash scripts, which iteratively connected the pulling code and energy minimization in GROMACS (70).

All simulations were run with the GROMACS 5.4.1 molecular dynamics engine^2^ with a time step of 2 fs. The LINCS algorithm (74) was applied to constrain all bond lengths with a relative geometric tolerance of 10^−4^. In line with its original parameterization, short-range interactions (van der Waals and Coulomb) were calculated using a cutoff scheme of 0.9 nm, which were evaluated based on a pair list recalculated every 5 time steps. Long-range interactions were handled using a reaction field (75) correction with a permittivity dielectric constant of 66. After initial setup, each system was energy minimized using 3,000 steps of conjugated gradient, followed by a thermal equilibration of 1 ns. A harmonic potential of 1,000 kJ mol^−2^, along the Z vector connecting the center of mass (COM) of the drug and the OM of the membrane, was applied in order to maintain the relative position of the drug with respect to each of the seven different regions of the membrane as described in the system setup section. During equilibration, bilayers were coupled to 1.0 bar using a Berendsen barostat (76) through a semi-isotropic approach with a relaxation time of 1.0 ps. Afterwards, production runs were coupled using a Parrinello barostat (77) algorithm, and a constant temperature of 310 K was maintained by weak coupling of the solvent and solute separately to a velocity-rescaling (78) scheme with a relaxation time of 1.0 ps. Production simulations were run for 20 ns, and trajectories were saved each 20 ps.

A total of 4,207 (∼84 µs) trajectories were analyzed using an in-home-developed bash script, which was directly interconnected to the in-built GROMACS tools.

### Statistical and machine learning methods.

Figure S2 shows an outline of the developed algorithm. There are two phases to feature selection. In the first phase, we employ a single sparse LASSO fit (using the cvglmnet function in the glmnet_python package), with a regularization parameter tuned to retain 50% ± 2% of the total descriptors in the batch of descriptors considered. In the second phase, using the retained ∼50% of the descriptors, we run two further (nonsparse) regressions, employing shuffling of the data along with 5-fold cross-validation to assess the robustness of the coefficients that result for each descriptor in a simple binomial model. We retain at most one descriptor from each cluster computed by correlation clustering of the descriptor (sub)set. We choose to retain the descriptor with the largest ratio of average coefficient divided by standard deviation of coefficient, as we expect that to be the most consistent and hence most generalizable representative of this cluster. Finally, we refit in an identical manner using the cluster representatives and discard any descriptor with an average that is within a standard deviation of zero as being unimportant. We run the second phase 100 times on different stratified subsets of the training data in order to perform a bootstrap analysis of the consistency with which specific descriptors are chosen.

The modeling experiments and parameters are summarized in Table S4. Seven different variables derived from the following experimental ratios were used for model outputs (Table S4): efflux = IC_50 PΔ6-Pore_/IC_50 PAO1-Pore_, permeation = IC_50 PΔ6-Pore_/IC_50 PΔ6_, EPI-1 = *g*(MIC_PAO1_/MPC_8 PA1032_), EPI-2 = *g*(MIC_PΔ6-Pore_/MPC_8 PA1032_), EPI_MPC_ = *g*(IC_50 PAO1_/MPC_8 PA1032_), and EPI_SS_ = SS_16 µM_/SS_0 µM_. SS_concn_ refers to the steady-state HT accumulation ratio at that concentration, and fold difference is the fold difference in HT fluorescence (16 µM/0 µM). The function *g* is a rescaling factor defined as where *xi* is the *i-*th entry in the ratio list and the MAX function is taken over the entire list.

For model fitting, we selected models created with the same number of total descriptors to avoid size effects. We employed up to the total number of descriptors retained during feature selection for the smallest subset (permeation). For final model fitting and assessment, we arrange the descriptors in order of the number of times they were chosen by the bootstrap phase of feature selection and then choose the top *N* descriptors, where *N* is the number returned by searching for a “gap” in the ordered descriptors by using the L method of Salvador and Chan (79) as a relatively conservative estimate that nonetheless does not retain descriptors in the tail of the distribution of the number of times they appeared in the bootstrap phase of feature selection. Once the final set of descriptors is selected, the LogisticRegressionCV class of the scikit-learn package was employed to learn a nonsparse binomial classifier employing the neg_log_loss scoring penalty with “balanced” class weight and an L2 penalty. The random state was arbitrarily set to 0 for consistency and ease of debugging.

In case of a 3-class multinomial regression classifier, feature selection was performed in a similar manner as for the binomial classifiers, except that in phase 1 we retain all descriptors that have a nonzero coefficient for any class, and in phase 2, we choose cluster representatives for all three classes. If two or more classes choose the same cluster representative, we retain it. Otherwise, we choose from the different class representatives randomly. Finally, we retain all descriptors for which at least for one class the average value is more than 1 standard deviation away from zero.

Model fitting is likewise performed in a similar manner, except that we employ an elasticnet penalty, which is a balance between L1 and L2 penalties that allows some of the descriptors to go to zero, in order to loosen the restrictions on descriptors relating to different classes, and we use the ovr, or one-vs-rest, formulation. Because of this, we use all top descriptors (76) returned from feature selection on the full set of all descriptors.

In order to assess the quality of the classifiers learned, we employed bootstrapping to estimate errors (Fig. S2). For each model, we fit on an arbitrarily chosen training set of 75% of the data using the train_test_split function of the scikit-learn package. We ensured that the class balance was the same for both this and the retained 25% testing set.
