# Supplementary figures and images for: Predictive Rules of Efflux Inhibition and Avoidance in Pseudomonas aeruginosa
Source: mBio. 2021 Jan 19;12(1):e02785-20. doi: 10.1128/mBio.02785-20 (PMC7845643; doi:10.1128/mBio.02785-20)

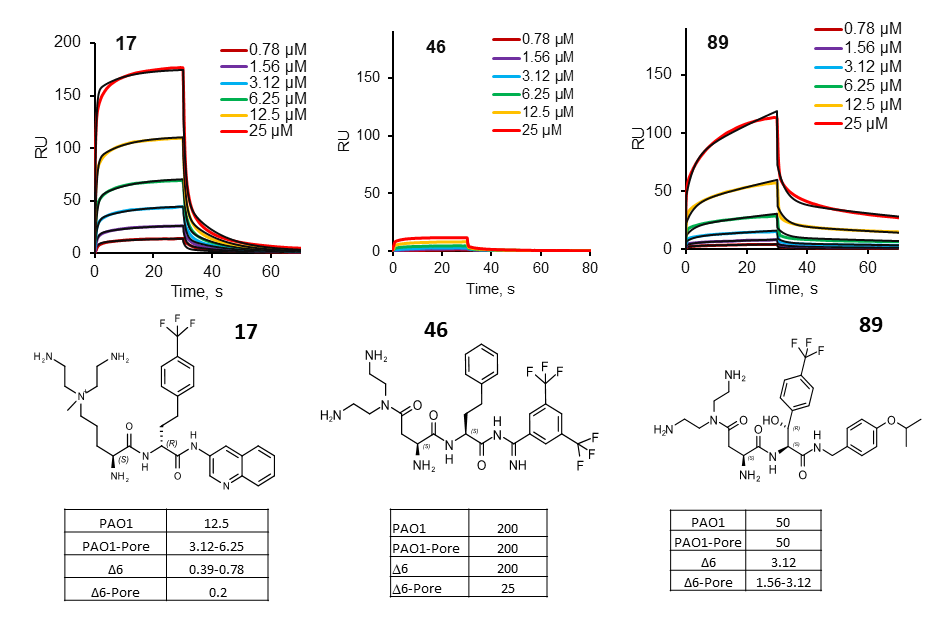

Supplement: FIG S1 [file mBio.02785-20-sf001.tif]

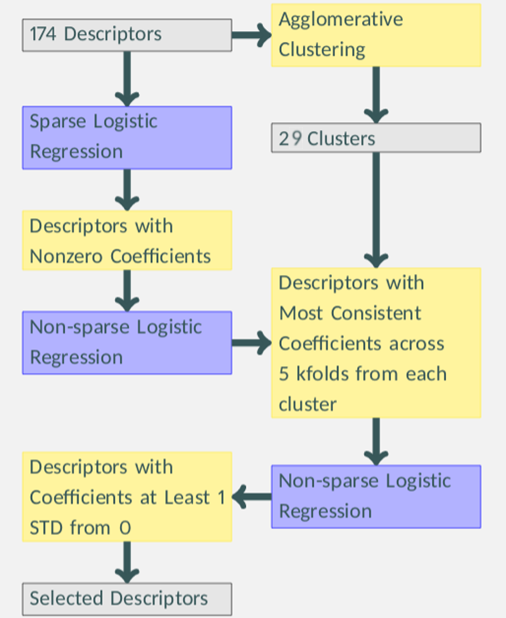

Supplement: FIG S2 [file mBio.02785-20-sf002.tif]

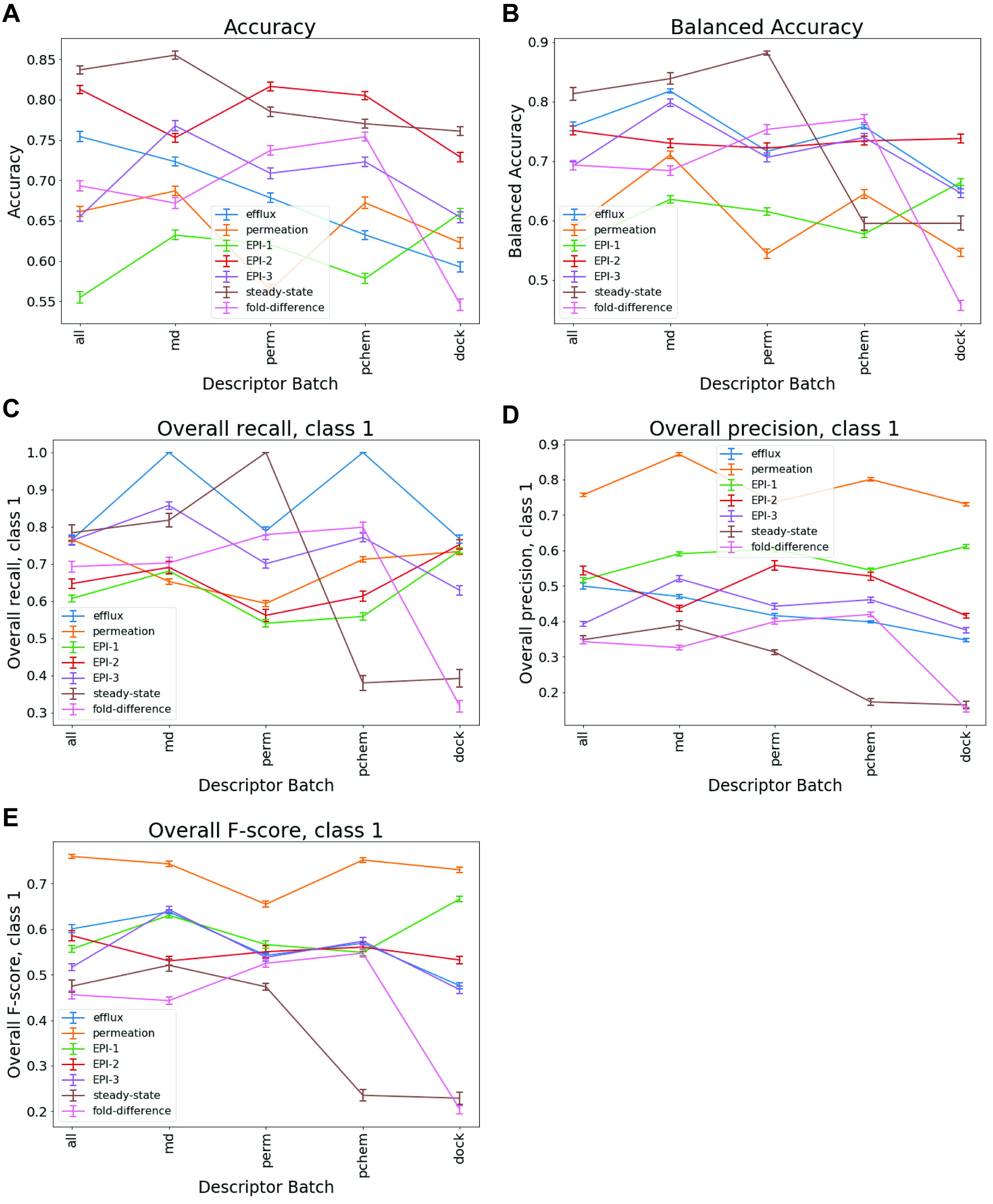

Supplement: FIG S3 [file mBio.02785-20-sf003.tif]

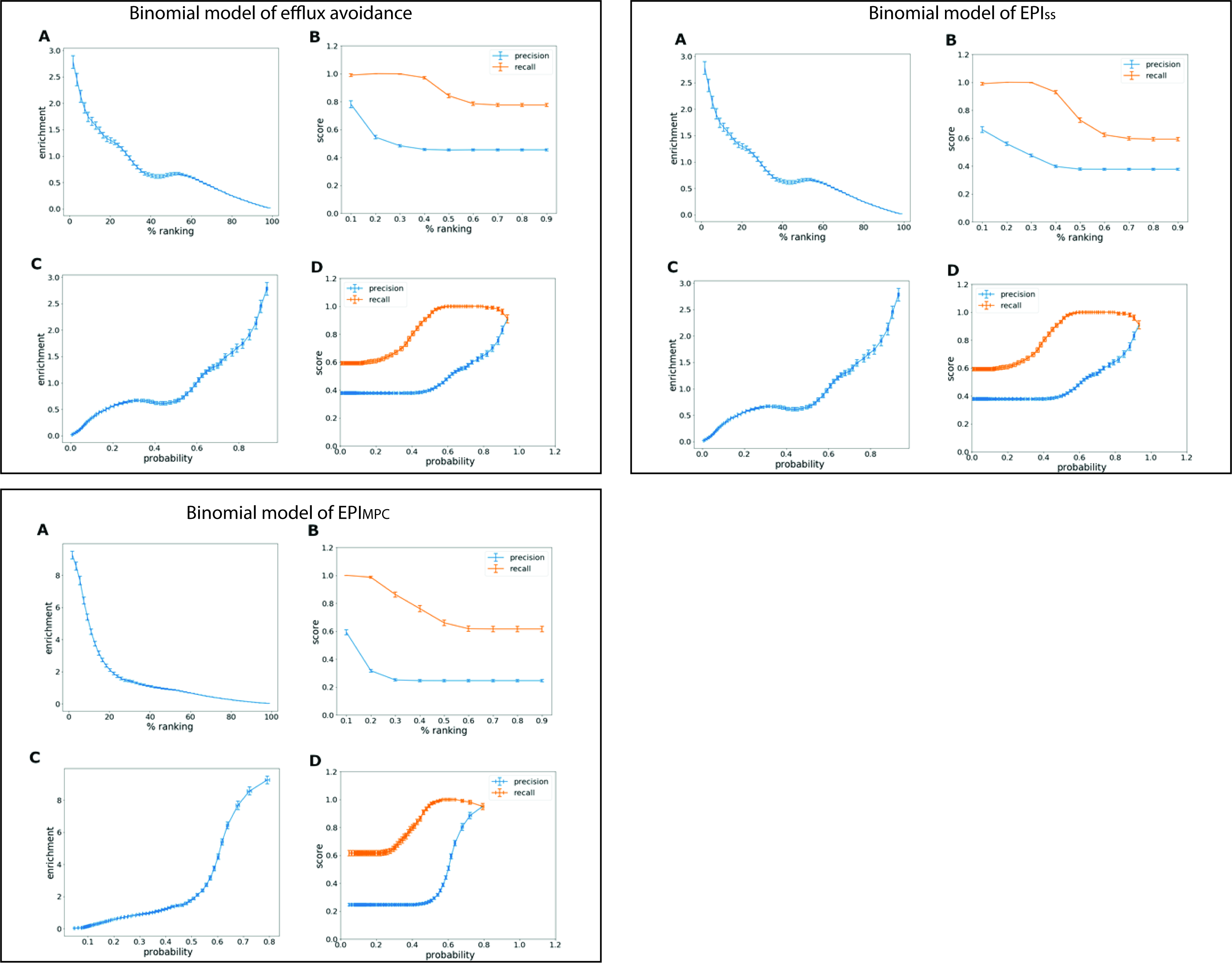

Supplement: FIG S4 [file mBio.02785-20-sf004.tif]
