# Supplementary material for: Predictive Rules of Efflux Inhibition and Avoidance in Pseudomonas aeruginosa
Source: mBio. 2021 Jan 19;12(1):e02785-20. doi: 10.1128/mBio.02785-20 (PMC7845643; doi:10.1128/mBio.02785-20)
Supplement: TEXT S1 [file mBio.02785-20-s0001.docx]

**Supplemental Methods**

***Binomial model assessment*.**

We assessed performance of the final models through the metrics of *enrichment,* precision and recall as a function of both probability and ranking. The *enrichment* of hits of a predictive model is a measure of how many more hits a model finds than would be found by a random search, in terms of a function derived from the model that ranks the hits. The *probability* is the probability assigned by the binomial model that a particular compound is a hit. The *ranking* is a more coarse-grained quantity. The ROC curve is a measure of the true positive rate (TPR) versus the false positive rate (FPR). When employing all descriptors to fit our models, we found that efflux, EPI_MPC_ and EPI_SS_ (see **Figure S4**) all have an enrichment that is monotonically decreasing when plotted versus ranking and monotonically increasing when plotted versus probability (lower *number* for ranking corresponds to *higher* probability, because the best possible rank is top 0%). This is a desirable behavior as it indicates that the higher the probability assigned by the model, the more likely it is that an item is truly a hit. Additionally, we observe excellent enrichment scores for high ranking/probability for efflux (maximum enrichment ~ 3), EPI_MPC_ (maximum enrichment ~ 3), and EPI_SS_ (maximum enrichment ~ 8), demonstrating that we are able to fit models that can substantially narrow experimental search spaces. We are unable to do the same for permeation (approximately monotonic but maximum enrichment only ~0.4), EPI-1 (non-monotonic, maximum enrichment ~0.4 at 0% ranking), EPI-2 (non-monotonic, maximum enrichment ~3 at ~20% ranking), or fold-difference (maximum enrichment ~1, at ~20% ranking), suggesting that greater noise in the experimental data corresponds to greater difficulty in fitting a predictive model.

Similar conclusions can be drawn from the precision and recall plots (**Figure S4**). Efflux, EPI_MPC_, and EPI_SS_ have monotonically increasing precision with probability. From these plots, we once again see that the classifiers behave sensibly for efflux, EPI_MPC_, and EPI_SS_ (the more stringent the probability cutoff, the better the performance) and also provide guidelines for how to interpret probability thresholds in terms of the expected performance of the classifier. For identifying compounds of interest for future experimental screening, one may employ the predictive models to assess their probabilities or the relative rankings of a group of such compounds. Setting a higher probability threshold for those compounds that are to be verified experimentally, or experimentally verifying them on at a time in order of probability ranking should improve the outcome.

***General synthetic methods.***

Unless otherwise noted, all materials were obtained from commercial vendors and used as is without further purification. All reactions were monitored by using thin-layer chromatography (TLC) on silica gel coated glass plates or by high-pressure liquid chromatography (HPLC: Ascentis express peptide ES C-18 column, OD 3cm X 4.6cm, 6min flow rate 1 mL/min; gradient = 95/5 → 5/95 CH_3_CN-H_2_O). Flash column chromatography was performed on a Combi-Flash ISCO with prepacked silica gel columns. ^1^H NMR spectra were recorded on a Bruker Avance II nuclear magnetic resonance spectrometer (400 MHz) with chemical shifts reported in δ ppm relative to DMSO-d_6_ or CDCl_3_ (s = singlet, d = doublet, t = triplet, m = multiplet, dd = doublet of doublets, etc). ^13^C NMR and ^19^F NMR spectra were obtained on the same instrument at 100 MHz and 376 MHz, respectively. Final compound purities were determined by HPLC using the conditions described above. High-resolution mass spectra were performed by College of Science Major Instrumentation Center, Old Dominion University, on a Bruker 12 Tesla APEX -Qe FTICR-MS with an Apollo II ion source.

*(2E)-3-(2-chlorophenyl)-N-[4-(4,5-dihydro-1H-imidazol-2-yl)phenyl]prop-2-enamide* **(SLUPP-225).** To a two-dram vial equipped with stir-bar, argon gas and ice bath, 2-Chlorocinnamic acid (46 mg, 0.25 mmol, 1 eq) was added and converted to the corresponding acid chloride via treatment with 2.0 M oxalyl chloride solution (1.0 mL) in dichloromethane (1.0 mL). A catalytic amount of DMF (2 drops) was added and the reaction was stirred overnight to completion at room temperature. The solvent was removed under reduced pressure, leaving 2-Chlorocinnamoyl chloride as a solid. In a separate vial 4-(4,5-Dihydro-1H-imidazol-2-yl)aniline (40 mg, 0.25 mmol, 1 eq) was dissolved in glacial acetic acid (1.5 mL), added to the acid chloride reaction vial and stirred overnight at room temperature. The solvent was removed under reduced pressure and the crude product was purified on a 50g C18 reversed-phase column (acetonitrile/water). The product was obtained as an impure TFA salt. The product was converted to free base in 4M NaOH (white solid, yield 16%). ^1^H NMR (400 MHz, DMSO-d_6_) δ 10.55 (s, 1H), 7.90 (d, *J*= 15.6 Hz, 1H), 7.79 (m, 5H), 7.57 (m, 1H), 7.45 (m 2H), 6.92 (d, *J*= 15.6 Hz, 1H), 3.62 (s, 4H). HRMS: m/z calculated for C_18_H_16_ClN_3_O [M + H]^+^ 326.1062; found [M + H]^+^ 326.1060.

*(2E)-N-[3,4-bis(4,5-dihydro-1H-imidazol-2-yl)phenyl]-3-(2-chlorophenyl)prop-2-enamide* **(SLUPP-778).** To a two-dram vial equipped with stir-bar, argon gas and ice bath, 2-Chlorocinnamic acid (46 mg, 0.25 mmol, 1 eq) was added and converted to the corresponding acid chloride via treatment with 2.0 M oxalyl chloride solution (1.0 mL) in dichloromethane (1.0 mL). A catalytic amount of DMF (2 drops) was added and the reaction was stirred overnight to completion at room temperature. The solvent was removed under reduced pressure, leaving 2-Chlorocinnamoyl chloride as a solid. In a separate vial 3,4-Bis(4,5-dihydro-1H-imidazol-2-yl)aniline (143 mg, 0.25 mmol, 1 eq) was dissolved in glacial acetic acid (1.5 mL), added to the acid chloride reaction vial and stirred overnight at room temperature. The solvent was removed under reduced pressure and the crude product was purified on a 50g C18 reversed-phase column (acetonitrile/water). The product was obtained as an impure TFA salt. The product was converted to free base in 4M NaOH (Tan solid, yield 27%). ^1^H NMR (400 MHz, DMSO-d_6_) δ 10.60 (s, 1H), 7.99 (d, *J* = 2.2 Hz, 1H), 7.90 (d, *J* = 15.7 Hz, 1H), 7.85 (dd, *J* = 8.6, 2.3 Hz, 1H), 7.81 – 7.76 (m, 1H), 7.66 (d, *J* = 8.5 Hz, 1H), 7.57 (dd, *J* = 6.0, 3.3 Hz, 1H), 7.46 (dd, *J* = 5.9, 3.5 Hz, 2H), 6.88 (d, *J* = 15.7 Hz, 1H), 3.59 (s, 8H). LCMS: m/z observed for C_21_H_20_ClN_5_O [M+H]^+^ 394.1.

*N-[3,4-bis(4,5-dihydro-1H-imidazol-2-yl)phenyl]-2-(4-chlorophenyl)acetamide* **(SLUPP-791).** To a two dram vial equipped with stir-bar, 3,4-Bis(4,5-dihydro-1H-imidazol-2-yl)aniline (143 mg, 0.25 mmol, 1 eq) was dissolved in glacial acetic acid (1.5 mL). 4-Chlorophenylacetyl chloride was added to the solution dropwise and stirred overnight at room temperature. The solvent was removed under reduced pressure and the crude product was purified on a 50g C18 reversed-phase column (acetonitrile/water). The product was obtained as an impure TFA salt. The product was converted to free base in 4M NaOH (Yellow solid, yield 13%). ^1^H NMR (400 MHz, DMSO-d_6_) δ 10.47 (s, 1H), 7.88 (d, *J* = 2.2 Hz, 1H), 7.71 (dd, *J* = 8.6, 2.2 Hz, 1H), 7.62 (d, *J* = 8.5 Hz, 1H), 7.44 – 7.31 (m, 4H), 3.67 (s, 2H), 3.57 (s, 7H). LCMS: m/z observed for C_20_H_20_ClN_5_O [M+H]^+^ 382.1.

*(2E)-N-[4-(4,5-dihydro-1H-imidazol-2-yl)phenyl]-3-[4-(propan-2-yl)phenyl]prop-2-enamide* **(SLUPP-417).** To a two-dram vial equipped with stir-bar, argon gas and ice bath, 4-Isopropylcinnamic acid (48 mg, 0.25 mmol, 1 eq) was added and converted to the corresponding acid chloride via treatment with 2.0 M oxalyl chloride solution (0.5 mL) in dichloromethane (1.5 mL). A catalytic amount of DMF (2 drops) was added and the reaction was stirred overnight to completion at room temperature. The solvent was removed under reduced pressure, leaving 4-Isopropylcinnamoyl chloride as a solid. In a separate vial 4-(4,5-Dihydro-1H-imidazol-2-yl)aniline (40 mg, 0.25 mmol, 1 eq) was dissolved in glacial acetic acid (1.5 mL), added to the acid chloride reaction vial and stirred overnight at room temperature. The solvent was removed under reduced pressure and the crude product was purified on a 50g C18 reversed-phase column (acetonitrile/water). The product was obtained as a TFA salt (yield 8%).^1^H NMR (400 MHz, DMSO-d_6_) δ 10.70 (s, 1H), 10.33 (s, 2H), 7.93 (d, *J* = 2.6 Hz, 3H), 7.63 (d, *J* = 15.9 Hz, 1H), 7.58 (d, *J* = 8.3 Hz, 2H), 7.34 (d, *J* = 8.4 Hz, 2H), 6.81 (d, *J* = 15.7 Hz, 1H), 3.99 (s, 4H), 1.22 (d, *J* = 6.9 Hz, 6H). LCMS: m/z observed for C_21_H_23_N_3_O [M+H]^+^ 334.1.

*(2E)-N-[3,4-bis(4,5-dihydro-1H-imidazol-2-yl)phenyl]-3-[4-(propan-2-yl)phenyl]prop-2-enamide* **(SLUPP-885).** To a two-dram vial equipped with stir-bar, argon gas and ice bath, 4-Isopropylcinnamic acid (48 mg, 0.25 mmol, 1 eq) was added and converted to the corresponding acid chloride via treatment with 2.0 M oxalyl chloride solution (1.0 mL) in dichloromethane (1.0 mL). A catalytic amount of DMF (2 drops) was added and the reaction was stirred overnight to completion at room temperature. The solvent was removed under reduced pressure, leaving 4-Isopropylcinnamoyl chloride as a solid. In a separate vial 3,4-Bis(4,5-dihydro-1H-imidazol-2-yl)aniline (143 mg, 0.25 mmol, 1 eq) was dissolved in glacial acetic acid (1.5 mL), added to the acid chloride reaction vial and stirred overnight at room temperature. The solvent was removed under reduced pressure and the crude product was purified on a 50g C18 reversed-phase column (acetonitrile/water). The product was obtained as a TFA salt (white solid, yield 9%). ^1^H NMR (400 MHz, DMSO-d_6_) δ 11.02 (s, 1H), 10.66 (s, 2H), 10.46 (s, 2H), 8.03 (dd, *J* = 8.7, 2.2 Hz, 1H), 7.86 (d, *J* = 8.6 Hz, 1H), 7.65 (d, *J* = 15.6 Hz, 1H), 7.59 (d, *J* = 8.3 Hz, 2H), 7.35 (d, *J* = 8.3 Hz, 2H), 6.84 (d, *J* = 15.7 Hz, 1H), 4.01 (d, *J* = 13.4 Hz, 8H), 1.22 (d, *J* = 6.9 Hz, 6H). LCMS: m/z observed for C_24_H_27_N_5_O [M+H]^+^ 402.2.

*6-bromo-N-[4-(4,5-dihydro-1H-imidazol-2-yl)phenyl]naphthalene-2-carboxamide* **(SLUPP416).**  To a two-dram vial equipped with stir-bar, argon gas and ice bath, 6-Bromo-2-naphthoic acid (63 mg, 0.25 mmol, 1 eq) was added and converted to the corresponding acid chloride via treatment with 2.0 M oxalyl chloride solution (0.5 mL) in dichloromethane (1.5 mL). A catalytic amount of DMF (2 drops) was added and the reaction was stirred overnight to completion at room temperature. The solvent was removed under reduced pressure, leaving 6-Bromo-2-naphthoyl chloride a solid. In a separate vial 4-(4,5-Dihydro-1H-imidazol-2-yl)aniline (40 mg, 0.25 mmol, 1 eq) was dissolved in glacial acetic acid (1.5 mL), added to the acid chloride reaction vial and stirred overnight at room temperature. The solvent was removed under reduced pressure and the crude product was purified on a 50g C18 reversed-phase column (acetonitrile/water). The product was obtained as a TFA salt (yellow solid, yield 12%). ^1^H NMR (400 MHz, DMSO-d_6_) δ 10.93 (s, 1H), 10.37 (s, 2H), 8.62 (s, 1H), 8.35 (d, *J* = 1.9 Hz, 1H), 8.08 (t, *J* = 5.7 Hz, 5H), 7.96 (d, *J* = 8.9 Hz, 2H), 7.78 (dd, *J* = 8.7, 2.0 Hz, 1H), 4.01 (s, 4H). LCMS: m/z observed for C_20_H_16_BrN_3_O [M]^+^ 394.0, [M+2H]^+^ 395.9.

*2N-[3,4-bis(4,5-dihydro-1H-imidazol-2-yl)phenyl]-6-bromonaphthalene-2-carboxamide* **(SLUPP-884).** To a two-dram vial equipped with stir-bar, argon gas and ice bath, 6-Bromo-2-naphthoic acid (63 mg, 0.25 mmol, 1 eq) was added and converted to the corresponding acid chloride via treatment with 2.0 M oxalyl chloride solution (1.0 mL) in dichloromethane (1.0 mL). A catalytic amount of DMF (2 drops) was added and the reaction was stirred overnight to completion at room temperature. The solvent was removed under reduced pressure, leaving 6-Bromo-2-naphthoyl chloride as a solid. In a separate vial 3,4-Bis(4,5-dihydro-1H-imidazol-2-yl)aniline (143 mg, 0.25 mmol, 1 eq) was dissolved in glacial acetic acid (1.5 mL), added to the acid chloride reaction vial and stirred overnight at room temperature. The solvent was removed under reduced pressure and the crude product was purified on a 50g C18 reversed-phase column (acetonitrile/water). The product was obtained as a TFA salt (Tan solid, yield 43%). ^1^H NMR (400 MHz, DMSO-d_6_) δ 11.21 (s, 1H), 10.72 (s, 2H), 10.55 (s, 2H), 8.65 (s, 1H), 8.56 (d, *J* = 2.1 Hz, 1H), 8.36 (d, *J* = 1.9 Hz, 1H), 8.22 (dd, *J* = 8.7, 2.1 Hz, 1H), 8.14 – 8.06 (m, 3H), 7.93 (d, *J* = 8.7 Hz, 1H), 7.79 (dd, *J* = 8.7, 2.0 Hz, 1H), 4.03 (d, *J* = 12.7 Hz, 8H). LCMS: m/z observed for C_23_H_20_BrN_5_O [M]^+^ 462.0, [M+2H]^+^464.0.

*N-(4-(4,5-dihydro-1H-imidazol-2-yl)phenyl)-6-(thiophen-3-yl)-2-naphthamide* **(SLUPP-1360).** Step 1: preparation of 6-(thiophen-3-yl)-2-naphthoic acid. 6-bromonaphthoic acid (0.5 g, 2 mmol, 1eq) was added to a 2-neck 50 mL RBF equipped with a stir bar and reflux condenser. 3-thienyl boronic acid (0.56 g, 4.4 mmol, 2.2 eq) was added to the RBF and the mixture was slurried in dioxane (20 mL). To this was added a 2.0M K_2_CO_3_-H_2_O solution (5 mL) which resulted in a homogeneous solution. The atmosphere was evacuated under vacuum and then back-filled with Argon. This was repeated two more times and then Pd(PPh_3_)_4_ (0.23g, 0.2 mmol, 0.1 eq) and the reaction was heated to 80 ^o^C overnight. The reaction was judged complete by LC-MS. Air was passed over the top of the reaction vessel to remove most of the dioxane. The reaction was then diluted with CH_2_Cl_2_ and the layers were separated. The aqueous layer was extracted with CH_2_Cl_2_ (3X). The organics were combined, dried and concentrated in vacuo. The crude material was purified by flash chromatography. Elution with hexanes-EtOAc (3:1 to 1:1) yielded the desired product (175 mg, 35% yield); LCMS: m/z observed for C_15_H_10_O_2_S[M+H]^+^ 255.1.

Step 2: Preparation of *N*-(4-(4,5-dihydro-1H-imidazol-2-yl)phenyl)-6-(thiophen-3-yl)-2-naphthamide. To a two-dram vial, fitted with a rubber septum, was added the above acid (0.075g, 0.29 mmol, 1 eq) which was dissolved in CH_2_Cl_2_ (1.5 mL) and cooled to 0 ^o^C with an ice bath. To this solution was then added a 2.0 M (COCl)_2_-CH_2_Cl_2_ (0.176 mL) and the resulting yellow solution was stirred at 0 ^o^C for 10 min and then allowed to warm to rt and stirred for 2h. The reaction was judged complete by LC-MS. The solvent was then removed in vacuo to give a yellow residue. The residue was then dissolved in HOAc (0.5 mL). In a separate vial, 4-(4,5-dihydro-1*H*-imidazol-2-yl)aniline (0.041g, 0.25 mmol, 1 eq) was dissolved in HOAc (1 mL) and allowed to stir at rt for 15 min. To this mixture was added the above acid chloride solution (0.5 mL) which resulted in a thick precipitate being formed. The mixture was stirred at rt for 2h. The reaction was then diluted in Et_2_O and filtered through a fritted funnel. The product was washed with water and more Et2O and dried. The resulting material was taken up in a small amount of DMSO and purified purified on a 50g C18 reversed-phase column (acetonitrile/water) to give the desired product (25mg, 20% yield) as a TFA salt. ^1^H NMR (400 MHz, DMSO-d_6_) δ 10.92 (s, 1H), 10.38 (Br s, 2H), 8.60 (s, 1H), 8.39 (s, 1H), 8.16-8.02 (m, 7H), 7.97 (d, J=8.6 Hz, 2H), 7.79-7.73 (m, 2H), 4.00 (S, 4H). LCMS: m/z observed for C_24_H_19_N_3_OS[M+H]^+^ 398.1.
