# Supplementary material for: Predictive Rules of Efflux Inhibition and Avoidance in Pseudomonas aeruginosa
Source: mBio. 2021 Jan 19;12(1):e02785-20. doi: 10.1128/mBio.02785-20 (PMC7845643; doi:10.1128/mBio.02785-20)
Supplement: TABLE S4 [file mBio.02785-20-st004.docx]

**Table S4.** Modeling experiments and parameters.

| **Experimental assay** | **Number of descriptors Employed** | **Number of times descriptors chosen by phase I** | **Descriptor set** |
| --- | --- | --- | --- |
| Efflux | 16 | 29 | All |
| Permeation | 14 | 38 |  |
| EPI-1 | 15 | 49 |  |
| EPI-2 | 16 | 38 |  |
| EPI_MPC_ | 16 | 26 |  |
| EPI_SS_ | 15 | 12 |  |
| Fold-difference | 14 | 25 |  |
| efflux | 16 | 20 | LigMexB |
| permeation | 14 | 56 |  |
| EPI-1 | 15 | 41 |  |
| EPI-2 | 16 | 37 |  |
| EPI_MPC_ | 16 | 40 |  |
| EPI_SS_ | 15 | 15 |  |
| Fold-difference | 14 | 60 |  |
| efflux | 16 | 7 | Permeation |
| permeation | 14 | 1 |  |
| EPI-1 | 15 | 10 |  |
| EPI-2 | 16 | 2 |  |
| EPI_MPC_ | 16 | 1 |  |
| EPI_SS_ | 15 | 1 |  |
| Fold-difference | 14 | 1 |  |
| efflux | 16 | 11 | Docking |
| permeation | 14 | 11 |  |
| EPI-1 | 15 | 9 |  |
| EPI-2 | 16 | 10 |  |
| EPI_MPC_ | 16 | 13 |  |
| EPI_SS_ | 15 | 20 |  |
| Fold-difference | 14 | 23 |  |
| efflux | 16 | 45 | Lig |
| permeation | 14 | 45 |  |
| EPI-1 | 15 | 43 |  |
| EPI-2 | 16 | 50 |  |
| EPI_MPC_ | 16 | 29 |  |
| EPI_SS_ | 15 | 14 |  |
| Fold-difference | 14 | 47 |  |
|  |  |  |  |
| Efflux-EPI-3 | 15 | 70 | All |
| Efflux-EPI-3 | 15 | 88 | LigMexB |
| Efflux-EPI-3 | 15 | 3 | Permeation |
| Efflux-EPI-3 | 15 | 8 | Docking |
| Efflux-EPI-3 | 15 | 52 | Lig |
