# Supplementary material for: Predictive Rules of Efflux Inhibition and Avoidance in Pseudomonas aeruginosa
Source: mBio. 2021 Jan 19;12(1):e02785-20. doi: 10.1128/mBio.02785-20 (PMC7845643; doi:10.1128/mBio.02785-20)
Supplement: TABLE S5 [file mBio.02785-20-st005.docx]

**Table S5.** Top ranked predicted efflux avoiders. CEF, cephalosporin, NA, not applied.

| **Database Name** | **Synonyms** | **Antibiotic class** | Probability | **MICs (mg/L) in strains:** | | | | |
| --- | --- | --- | --- | --- | --- | --- | --- | --- |
|  |  |  |  | **PAO1** | **PAO1-Pore** | | **Δ6** | **Δ6-Pore** |
| OU-31498 | carumonam | monobactam | 0.85 | ND | | ND | ND | ND |
| OU-672 | moxalactam | CEF | 0.83 | 62.5 | | 15.63 | 62.5 | 0.97 |
| OU-640 | ceftriaxone | CEF | 0.81 | 15.6 | | 0.97 | 3.9 | 0.06 |
| OU-666 | cefonicid | CEF | 0.81 | 62.5 | | 3.9 | 3.9 | 3.9 |
| OU-031452 | cefpiramide | CEF | 0.80 | >1000 | | >1000 | >1000 | 250 |
| OU-668 | ceftibuten | CEF | 0.80 | >2000 | | 2000 | 1000 | 128 |
| OU-31514 | tigemonam | monobactam | 0.79 | ND | | ND | ND | ND |
| OU-73 | SLU-862 | NA | 0.79 | >100 | | >100 | >100 | >100 |
| OU-654 | cefotetan | CEF | 0.78 | >400 | | 200 | 100 | 12.5 |
| OU-31511 | sulbenicillin | PEN | 0.77 | ND | | ND | ND | ND |
| OU-31496 | aztreonam | monobactam | 0.77 | ND | | ND | ND | ND |
| OU-235 | SLU-340 | NA | 0.77 | >100 | | >100 | >100 | >100 |
| OU-213 | SLU-368 | NA | 0.76 | >100 | | >100 | >100 | >100 |
| OU-634 | cefoperazone | CEF | 0.76 | 3.9 | | 0.97 | 0.97 | 0.97 |

**Table S5-Continued.** Top ranked predicted EPIs.

| **Database**  **Name** | **Synonyms** | **Probability** | **MICs (mg/L) in strains:** | | | | **SS_conc_ ratio** |
| --- | --- | --- | --- | --- | --- | --- | --- |
|  |  |  | **PAO1** | **PAO1-Pore** | **Δ6** | **Δ6-Pore** |  |
| OU-1256 | SLU-1190 | 0.87 | >200 | >200 | >200 | >200 | ND |
| OU-31469 | Basilea-1 | 0.85 | >100 | >100 | 50 | 50 | 4.49 |
| OU-31471 | Basilea-2 | 0.84 | 100 | 100 | 25 | 25 | 7.20 |
| OU-31472 | Basilea-3 | 0.82 | >100 | >100 | 100 | 100 | 4.62 |
| OU-31453 | orbifloxacin | 0.80 | 1.56 | 0.39 | 0.097 | 0.0061 | ND |
| OU-618 | sarafloxacin | 0.80 | 0.39 | 0.39 | 0.024 | 0.0061 | ND |
| OU-1362 | NA | 0.80 | ND | ND | ND | ND | ND |
| OU-235 | SLU-340 | 0.77 | >100 | >100 | >100 | >100 | ND |
| OU-153 | SLU-579 | 0.76 | >100 | >100 | >100 | 50 | ND |
| OU-1140 | Z407305386 | 0.75 | >100 | >100 | >100 | >100 | ND |
| OU-789 | Norfloxacin | 0.75 | 1 | 1 | 0.063 | 0.016 | ND |
